# Removal of PCR Error Products and Unincorporated Primers by Metal-Chelate Affinity Chromatography

**DOI:** 10.1371/journal.pone.0014512

**Published:** 2011-01-14

**Authors:** Indhu Kanakaraj, David L. Jewell, Jason C. Murphy, George E. Fox, Richard C. Willson

**Affiliations:** 1 Department of Biology and Biochemistry, University of Houston, Houston, Texas, United States of America; 2 Department of Chemical and Biomolecular Engineering, University of Houston, Houston, Texas, United States of America; University of Southampton, United Kingdom

## Abstract

Immobilized Metal Affinity Chromatography (IMAC) has been used for decades to purify proteins on the basis of amino acid content, especially surface-exposed histidines and “histidine tags” genetically added to recombinant proteins. We and others have extended the use of IMAC to purification of nucleic acids via interactions with the nucleotide bases, especially purines, of single-stranded RNA and DNA. We also have demonstrated the purification of plasmid DNA from contaminating genomic DNA by IMAC capture of selectively-denatured genomic DNA. Here we describe an efficient method of purifying PCR products by specifically removing error products, excess primers, and unincorporated dNTPs from PCR product mixtures using flow-through metal-chelate affinity adsorption. By flowing a PCR product mixture through a Cu^2+^-iminodiacetic acid (IDA) agarose spin column, 94–99% of the dNTPs and nearly all the primers can be removed. Many of the error products commonly formed by Taq polymerase also are removed. Sequencing of the IMAC-processed PCR product gave base-calling accuracy comparable to that obtained with a commercial PCR product purification method. The results show that IMAC matrices (specifically Cu^2+^-IDA agarose) can be used for the purification of PCR products. Due to the generality of the base-specific mechanism of adsorption, IMAC matrices may also be used in the purification of oligonucleotides, cDNA, mRNA and micro RNAs.

## Introduction

Current methods of PCR product purification include electrophoretic separation, enzymatic degradation of single-stranded DNA with Exonuclease I and Shrimp Alkaline Phosphatase, and adsorption on silica matrices. Smith et al. have demonstrated the removal of mismatch sequences from PCR products using mutHLS proteins [1]. These methods can be expensive, time consuming, and/or difficult to automate. Enzyme-based techniques also can be complicated by the dependence of enzymatic activity on storage and reaction conditions.

We have developed a technique for removing PCR error products, unincorporated primers and dNTPs from a PCR product mixture using immobilized metal affinity chromatography (IMAC). IMAC is widely employed for the purification of both native and histidine-tagged recombinant proteins [2]. Transition metal ions such as Cu^2+^, Ni^2+^, and Zn^2+^ immobilized on chelators such as iminodiacetic acid (IDA) and nitrilotriacetic acid (NTA) have been shown to interact with histidine and tryptophan residues [3]. The purine bases of DNA/RNA contain aromatic and imidazolyl nitrogens similar to those of histidine and tryptophan. These aromatic nitrogens are shielded in double-stranded DNA, but are free and sterically-accessible in dNTPs, single-stranded DNA, mRNA and micro RNA. We previously speculated that IMAC could be extended from protein purification to the purification of nucleic acids, by separating nucleotides and single-stranded DNA/RNA from double-stranded DNA. In fact, mononucleotides were long ago shown to bind to IMAC matrices [4], [5]. We subsequently showed that metal-chelate binding can separate single-stranded RNA from double-stranded DNA by adsorption, or by selective precipitation with “smart” polymers [6], [7] and can be used in the separation of genomic DNA from plasmid DNA with selective renaturation [8]. We also found that the addition of neutral osmolytes enhances the binding of RNA and oligonucleotides to metal-chelate matrices [9], raising the possibility of water elution of nucleic acids from IMAC adsorbents under-loaded with metal, creating a repulsive surface charge through the unloaded anionic chelators [10]. Tan et al. recently reported that a combination of IMAC and metal affinity precipitation can be used to purify plasmid DNA by removing RNA and endotoxin [11], [12], and Luo et al. used IMAC to remove endotoxin from biological solutions [13]. Nastasijevic et al. demonstrated the use of a PCR-added dA_20_ tail as an affinity tag for capturing double-stranded PCR products on metal-chelate matrices and also proposed a method of IMAC purification of polyA-tailed mRNA [14]. In this work, we describe the purification of PCR products using Cu^2+^-IDA spin columns, with substantial reduction of erroneous PCR products, unincorporated primers and dNTPs.

## Results and Discussion

The use of a spin column allows the efficient use of small amounts of adsorbent and sample. The columns can be equilibrated with the appropriate buffer in a few minutes with a few centrifugation steps. With the appropriate equipment, e.g., filter-bottom plates, most steps are automatable.

**Figure 1:**
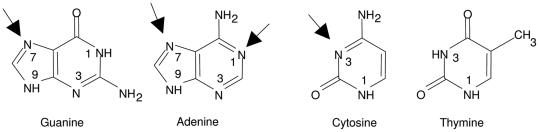
Metal ion binding sites of the DNA bases.

Previously, it was determined that single-stranded DNA binds to IMAC adsorbents whereas double-stranded DNA does not [6], [11], [12], [14]. Also, we have previously reported that mismatches in a heteroduplex oligonucleotide were captured by IMAC, and that single-stranded oligonucleotides were readily separated from double-stranded DNA [6]. However, the base content of an oligonucleotide is known to have a strong influence on its ability to bind to metal-chelate adsorbents. The purines, adenine and guanine, have a strong affinity for the Ni-IDA matrix, while the pyrimidines, cytosine and thymine, have a much weaker affinity [6]. These results can be understood by reference to X-ray crystallographic studies [15] of nucleotide-metal ion binding sites (Figure 1). Adenine, with its two metal binding sites, should show the best affinity, whereas thymine, with no strong metal binding sites, should show little or no affinity. While guanine and cytosine each have one binding site, the guanine site is less sterically-hindered than that of cytosine. Consistent with these structural results, it was found that guanine has greater affinity for an immobilized metal than cytosine [6]. Thus, it can be expected that an oligonucleotide (e.g., a PCR primer) with typical purine content should be readily separable from double-stranded DNA (though primers with very low purine content might not be well resolved from dsDNA).

### Removal of Primers and Error Products from a PCR Product Mixture

**Figure 2:**
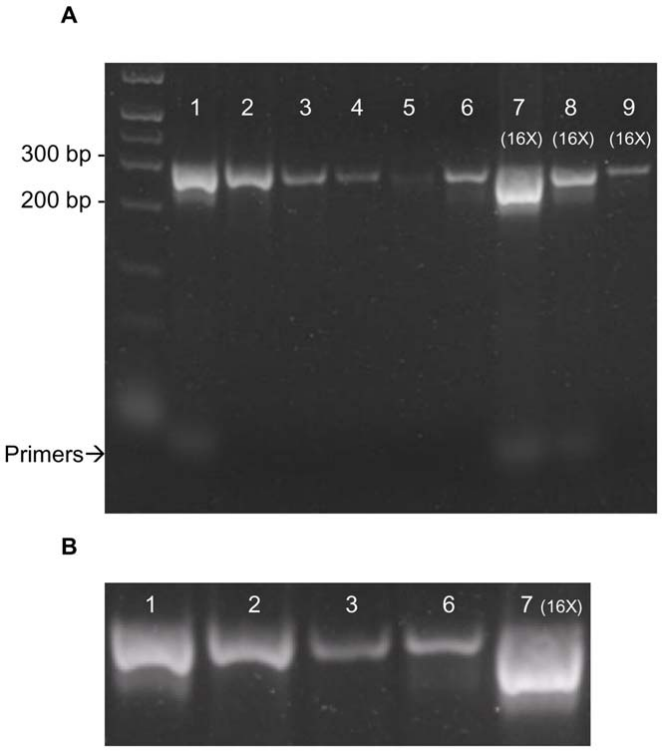
Cu^2+^-IMAC purification of PCR product mixture from amplifying a region of lambda bacteriophage genomic DNA. (**A**) Gel picture showing Cu^2+^-IMAC purification of PCR product mixture from amplification of a 280 bp region of lambda bacteriophage genomic DNA. Lane 1: 5 µL of unpurified PCR product; Lane 2: 5 µL of purified PCR product, flow-through after direct application of PCR product mixture to Cu^2+^-IMAC column; Lanes 3–5: 5 µL of consecutive 20 µL column washes with 250 mM NaCl, 20 mM HEPES, pH 7.0; Lane 6: 5 µL of first elution with 20 µL 500 mM imidazole in 250 mM NaCl, 20 mM HEPES, pH 7.0; Lanes 7–9: 5 µL of first, second and third elutions, respectively with 20 µL 500 mM imidazole in 250 mM NaCl, 20 mM HEPES, pH 7.0. Lanes 7–9 were concentrated 16-fold by ethanol precipitation to enhance sensitivity. (**B**) Expanded views of 280 bp product from selected lanes of Figure 2A. Lane 1: Unpurified PCR product, corresponding to Lane 1 of Figure 2A; Lane 2: Purified flow-through PCR product, corresponding to Lane 2 of Figure 2A; Lane 3: First wash with 250 mM NaCl, 20 mM HEPES, pH 7.0, corresponding to Lane 3 of Figure 2A; Lane 6: First elution with 500 mM imidazole in 250 mM NaCl, 20 mM HEPES, pH 7.0, corresponding to Lane 6 of Figure 2A; Lane 7: 16 times concentrated product of first imidazole elution, corresponding to Lane 7 of Figure 2A.

20 µL of a *Taq* PCR reaction product mixture from a lambda bacteriophage genomic DNA template was applied to each of two 125 µL Cu^2+^-IDA IMAC spin columns. The pooled flow-through product from two identical columns was loaded onto a 1.2% agarose SB (sodium boric acid) gel stained with SYBR Gold Nucleic Acid Gel Stain (Molecular Probes, Eugene, OR, USA) for analysis by electrophoresis. Figure 2A, lane 2 shows the purified PCR product; no primer band is visible in the product, indicating that both forward and reverse primers have been removed. Figure 2B, lane 2 also illustrates the removal of the higher-mobility error products from the product mixture. The first column was then washed with three consecutive 20 µL aliquots of non-eluting buffer A. Each wash was collected and analyzed on the same gel (Figure 2A, lanes 3–5) showing that additional fractions of pure double-stranded product can be obtained if desired. To characterize the contaminants captured by IMAC, 20 µL of eluent (500 mM imidazole in buffer A) was added to the second column and incubated for 15 min, then centrifuged for 2 min at 1100 xg. For the 2^nd^ and 3^rd^ elutions, 20 µL of eluent was added and centrifuged for 2 min at 1100 xg (no incubation). The primer is eluted from the column in the first two elutions, along with more double-stranded product (Figure 2A, lanes 6–9). In Figure 2A, lanes 7–9 were concentrated 16-fold by ethanol precipitation to enhance sensitivity. Eluates from four Cu^2+^-IMAC columns were pooled together (total volume 80 µL), and DNA precipitated using 1/10 volume of 3 M potassium acetate, pH 5.0 and 2.5 volumes of absolute ethanol. The pellet was washed with 250 µL of 70% ethanol, air dried and then dissolved in 5 µL of water and loaded into the gel.

In addition to the primers, in the concentrated eluates a smear of Taq PCR error products can be seen below the 280 bp product (Figure 2B, lane 7); these are effectively removed by IMAC (Figure 2B, lane 2). Error fragments are commonly produced in *Taq* PCR by insertion of mismatched bases [16]. *Taq* has difficulty extending the DNA chain past a mismatch, resulting in sticky-ended products [17], which have sufficient single-stranded regions to bind the Cu^2+^ column. Similar results as shown for lambda DNA were obtained for removal of PCR error products from products of PCR amplification of a 1.1 kbp D-alanine-D-alanine ligase A gene from *E.coli* genomic DNA (data shown in Figure S1).

### Removal of dNTPs from a PCR Product Mixture

Each dNTP (as 60 µL of a 100 µM solution) was loaded onto triplicate 100 µL Cu^2+^ spin columns, which were processed as described above. The flow-through was collected from each column and analyzed on a UV spectrophotometer. Approximately 99% of the dATP and dGTP was removed - 99±0.5% (0.90±0.00 absorbance) and 99±0.2% (1.50±0.01 absorbance), respectively. The deoxy pyrimidine triphosphates dCTP and dTTP bound to the column less stringently - 95±2% (0.88±0.02 absorbance) and 94±1% (1.36±0.00 absorbance) bound, respectively. In each case, the concentration of dNTPs was reduced from 100 µM to less than 6 µM, a level that should not inhibit subsequent sequencing or other enzymatic reactions [18].

### Sequencing of IMAC-processed PCR Mixtures

Contaminants such as excess salts, PCR primers, and dNTPs have a detrimental effect on sequencing of PCR products. In the sequencing reaction, primers and nucleotides will compete with the sequencing primer and ddNTPs. This results in additional products which, when analyzed on the sequencing gel, make it difficult or impossible to determine the correct bases, causing a “no-call”, the number of which can be used to judge the relative quality of a PCR product. Phred quality scores also can be used to assess the quality of the sequence, with a Phred quality score of 20 implying a 1-in-100 probability that the base is incorrectly assigned [19], [20].

**Figure 3:**
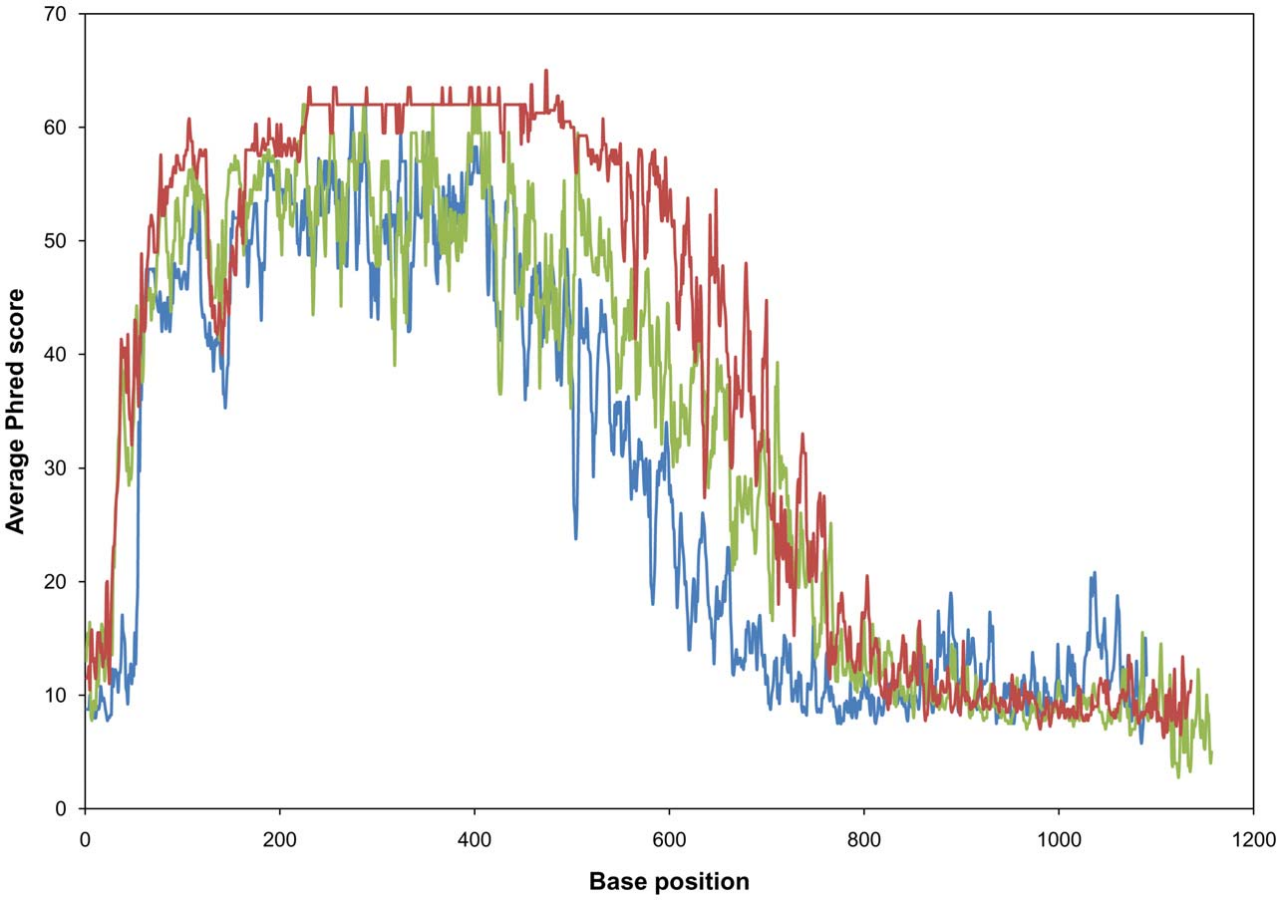
Average Phred score (using NT primer) versus base position. IMAC purified (red) and QIAquick purified (green) samples have better quality scores than unpurified (blue) samples.

SeqWright (Houston, TX, USA) performed dideoxynucleotide sequencing reactions on three sets of samples – the products of PCR amplification of a 1.1 kbp D-alanine-D-alanine ligase A encoding region of the *E.coli* genomic DNA, this PCR product after purification by IMAC, and this PCR product purified using the QIAquick PCR Purification kit (Qiagen, Valencia, CA, USA). Four independent PCR reaction samples were sequenced after each treatment, for a total of 12. Each product was sequenced from each end (using primers designated CT and NT), for a total of 24 reactions. Sequencing used BigDyes™ ddNTPs (Applied Biosystems, Foster City, CA, USA), according to the manufacturer's directions. The average no-calls ‘N’s observed after 10 correctly called consecutive bases in the first 800 bases for unpurified PCR products, QIAquick purified and IMAC purified samples were 38±24, 2±2 and 7±4 respectively using CT primer (Table S1) and 73±38, 4±2 and 14±8 respectively using NT primer (Table S2). Raw sequence traces are provided in Figure S2 - CT primer and Figure S3 - NT primer and ClustalW alignment of the sequences are shown in Figure S4. Also, Phred quality scores for each base were provided by SeqWright. The numbers of Phred 20 bases for unpurified PCR products, QIAquick purified and IMAC purified samples were 590±79, 795±13 and 815±29 respectively using CT primer and 596±45, 713±14 and 732±28 respectively using NT primer (Table 1). Figure 3, showing a plot of the average Phred score versus base position obtained using the NT primer (and a plot of the results using the CT primer shown in Figure S5), demonstrates that the sequence quality is greatly improved for IMAC purified samples compared to that of unpurified samples, and is at least comparable to the quality observed for QIAquick purified samples. These results show that unoptimized IMAC purification can yield purified product comparable in quality to that obtained using the commercial silica gel membrane QIAquick PCR Purification kit.

**Table 1:** Number of Phred 20 bases obtained after various treatments.

	CT Primer			NT Primer		
Sample	Unpurified PCR	QIAquick purified PCR	IMAC purified PCR	Unpurified PCR	QIAquick purified PCR	IMAC purified PCR
1	539	787	826	638	699	704
2	526	795	800	630	704	714
3	699	814	851	549	726	763
4	597	785	784	565	726	748
Mean±SD	590±79	795±13	815±29	596±45	713±14	732±28

From these results, it is clear that IMAC can remove primers, error products and dNTPs from PCR product mixtures, suggesting that IMAC (specifically Cu^2+^-IDA) is a promising approach to the purification of PCR products.

IMAC purification might also improve the results of PCR product cloning, and sequencing by non-Sanger methods. In addition to purifying double-stranded PCR products, IMAC should be applicable to the purification of double-stranded DNA amplicons generated by other *in vitro* DNA amplification methods such as Loop mediated isothermal amplification (LAMP), Helicase-dependent amplification (HDA) and Multiple displacement amplification (MDA). These results also suggest the application of IMAC in the purification of cDNAs, mRNAs and microRNAs.

## Materials and Methods

A 280 bp region of lambda bacteriophage genomic DNA (USB Corporation, Cleveland, OH, USA) was amplified in 50 µL PCR reactions containing 2 pg of lambda genomic DNA template, 1X PCR buffer, 200 µM each dNTP, 1 U Go*Taq* Flexi DNA Polymerase (Promega, Madison, WI, USA), 1.3 mM magnesium chloride and 2 µM each of the primers, 5′-GGCTTCGGTCCCTTCTGT-3′ and 5′-CACCACCTGTTCAAACTCTGC-3′. The reactions were carried out in a MJ Mini Personal thermal cycler (Bio-Rad, Hercules, CA, USA) as follows: 35 cycles of 45 s at 95°C (denaturation), 45 s at 55°C (annealing), 45 s at 72°C (elongation), followed by a final elongation step at 72°C for 5 min.

For other experiments, an 1.1 kbp D-alanine-D-alanine ligase A encoding region of the *E.coli* genomic DNA was amplified in 50 µL PCR reactions containing 10 ng of the genomic DNA, 1X PCR buffer, 200 µM each dNTP, 1 U Go*Taq* Flexi DNA Polymerase (Promega, Madison, WI, USA), 1 mM Magnesium chloride and 1 µM each of the primers, 5′- GGGGCATATGGAAAAACTGCGGGTAGGAATCG -3′ (NT primer) and 5′- GGGGGGATCCGGGCGTTAAAATATTACATTGTGGTT -3′ (CT primer). The reactions were carried out in a MJ Mini Personal thermal cycler (Bio-Rad, Hercules, CA, USA) as follows: 30 cycles of 45 s at 95°C (denaturation), 1 min at 66.5°C (annealing), 1 min at 72°C (elongation), followed by a final elongation step at 72°C for 5 min.

### Adsorbent Preparation

2 mL of Chelating Sepharose Fast Flow (GE Healthcare, Piscataway, NJ, USA) in 20% ethanol was added to a 2 mL polypropylene tube. The tube was centrifuged in a tabletop microcentrifuge at 11,000 xg for 2 min, the supernatant was decanted, and the beads were washed three times with DI H_2_O to remove residual ethanol. Then, 0.6 mL of 50 mM CuCl_2_ was added and the tube was mixed end-over-end on a Cole-Parmer rotor for 15 minutes. Finally, the beads were washed three times with 20 mM HEPES, 250 mM NaCl, pH 7.0 (“Buffer A”) to remove excess unchelated metal ions and to equilibrate the matrix.

### Spin Column Preparation and Use

Using a 1 ml pipette tip with the bottom quarter cut off to accommodate the *ca.* 90 µm adsorbent particles, 250 µL of vortex-suspended metal-charged matrix slurry (125 µL buffer A, 125 µL wet adsorbent) was added to a Micro Bio-Spin Chromatography column (Bio-Rad, Hercules, CA, USA). The column was then inserted into a 2 mL polypropylene microcentrifuge tube and centrifuged for 2 min at 1100 xg in a tabletop microcentrifuge, leaving 125 µL of packed wet beads.

The receiving microcentrifuge tube was replaced with a fresh one. Then, 20 µL of the PCR mixture was added to the prepared spin column and after allowing 15 min for adsorption, the column was centrifuged for 2 min at 1100 xg. The PCR mixture passes through the column and is depleted of primers, nucleotides and error products, producing purified double-stranded product. Some double-stranded product is trapped in the interstitial space of the column and can be recovered if desired by washing the column with non-eluting buffer A.

## Supporting Information

Figure S1 Cu^2+^-IMAC purification of PCR product mixture from amplifying a region of *E.coli* genomic DNA. Lane 1: normal loading (0.5 µL) of unpurified PCR product; Lane 2: overloading (4 µL) of unpurified PCR product; Lane 3: normal loading (0.5 µL) of purified PCR product, flow-through after direct application of PCR product mixture to Cu^2+^-IMAC column; Lane 4: overloading(4 µL) of purified PCR product Lanes 5-7: 4 µL of consecutive 20 µL column washes with 250 mM NaCl, 20 mM HEPES, pH 7.0; Lane 8: 4 µL of first elution with 20 µL 500 mM imidazole in 250 mM NaCl, 20 mM HEPES, pH 7.0; Lanes 9-11: 4 µL of first, second and third elutions, respectively with 20 µL 500 mM imidazole in 250 mM NaCl, 20 mM HEPES, pH 7.0. Lanes 9-11 were concentrated 10-fold by ethanol precipitation to enhance sensitivity.
(1.42 MB TIF)

Figure S2 Raw sequence trace using CT primer.
(5.44 MB PDF)

Figure S3 Raw sequence trace using NT primer.
(5.62 MB PDF)

Figure S4 ClustalW alignment of sequence data generated using NT and CT Primers.
(0.08 MB DOC)

Figure S5 Average Phred score (using CT primer) versus base position. IMAC purified (red) and QIAquick purified (green) samples have better quality scores than unpurified (blue) samples.
(0.36 MB PDF)

Table S1 Number of no-calls using CT primer.
(0.03 MB DOC)

Table S2 Number of no-calls using NT primer.
(0.03 MB DOC)
