# Supplementary figures and images for: Removal of PCR Error Products and Unincorporated Primers by Metal-Chelate Affinity Chromatography
Source: PLoS One. 2011 Jan 14;6(1):e14512. doi: 10.1371/journal.pone.0014512 (PMC3021510; doi:10.1371/journal.pone.0014512)

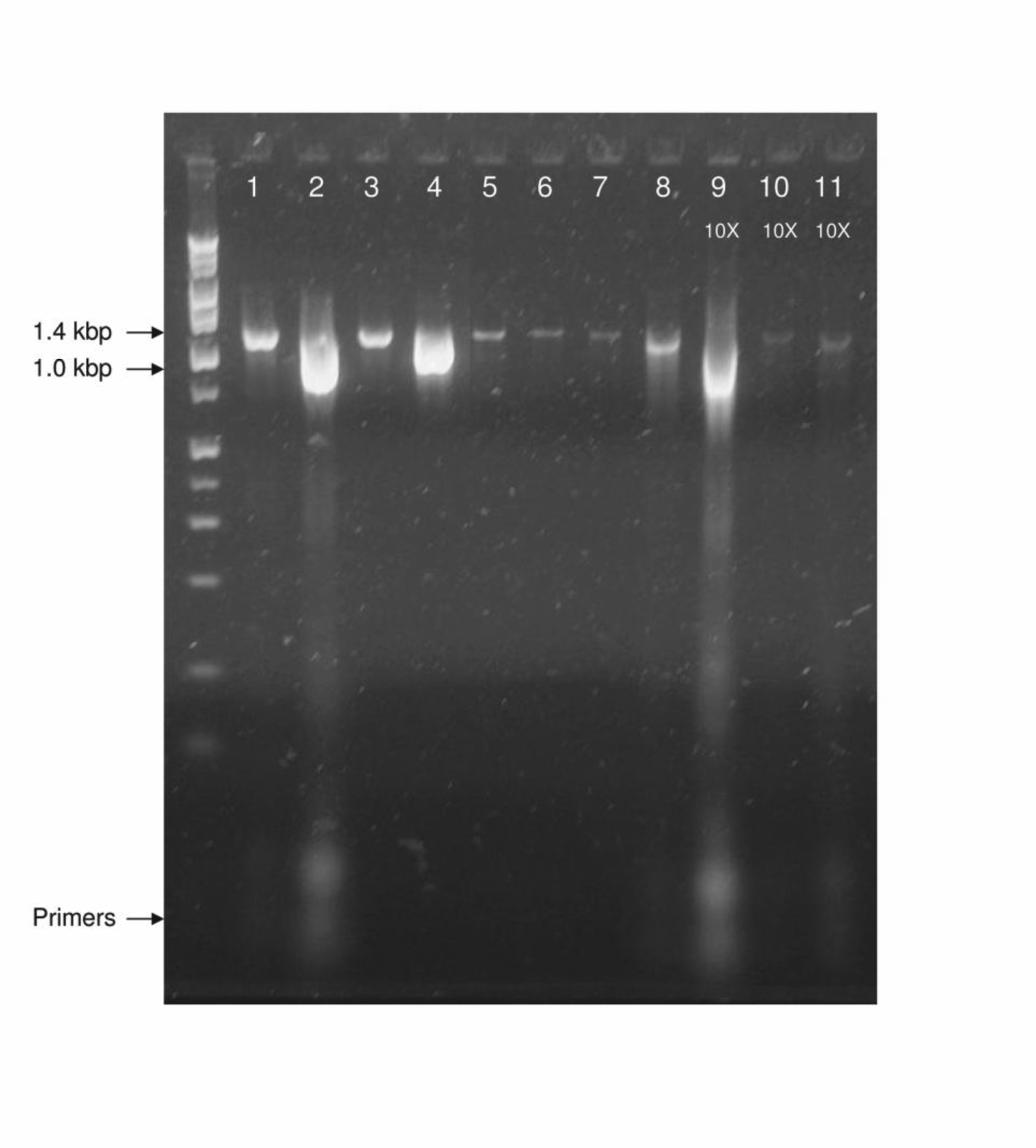

Supplement: Figure S1 — Cu2+-IMAC purification of PCR product mixture from amplifying a region of E.coli genomic DNA. Lane 1: normal loading (0.5 µL) of unpurified PCR product; Lane 2: overloading (4 µL) of unpurified PCR product; Lane 3: normal loading (0.5 µL) of purified PCR product, flow-through after direct application of PCR product mixture to Cu2+-IMAC column; Lane 4: overloading(4 µL) of purified PCR product Lanes 5-7: 4 µL of consecutive 20 µL column washes with 250 mM NaCl, 20 mM HEPES, pH 7.0; Lane 8: 4 µL of first elution with 20 µL 500 mM imidazole in 250 mM NaCl, 20 mM HEPES, pH 7.0; Lanes 9-11: 4 µL of first, second and third elutions, respectively with 20 µL 500 mM imidazole in 250 mM NaCl, 20 mM HEPES, pH 7.0. Lanes 9-11 were concentrated 10-fold by ethanol precipitation to enhance sensitivity. (1.42 MB TIF) [file pone.0014512.s001.tif]

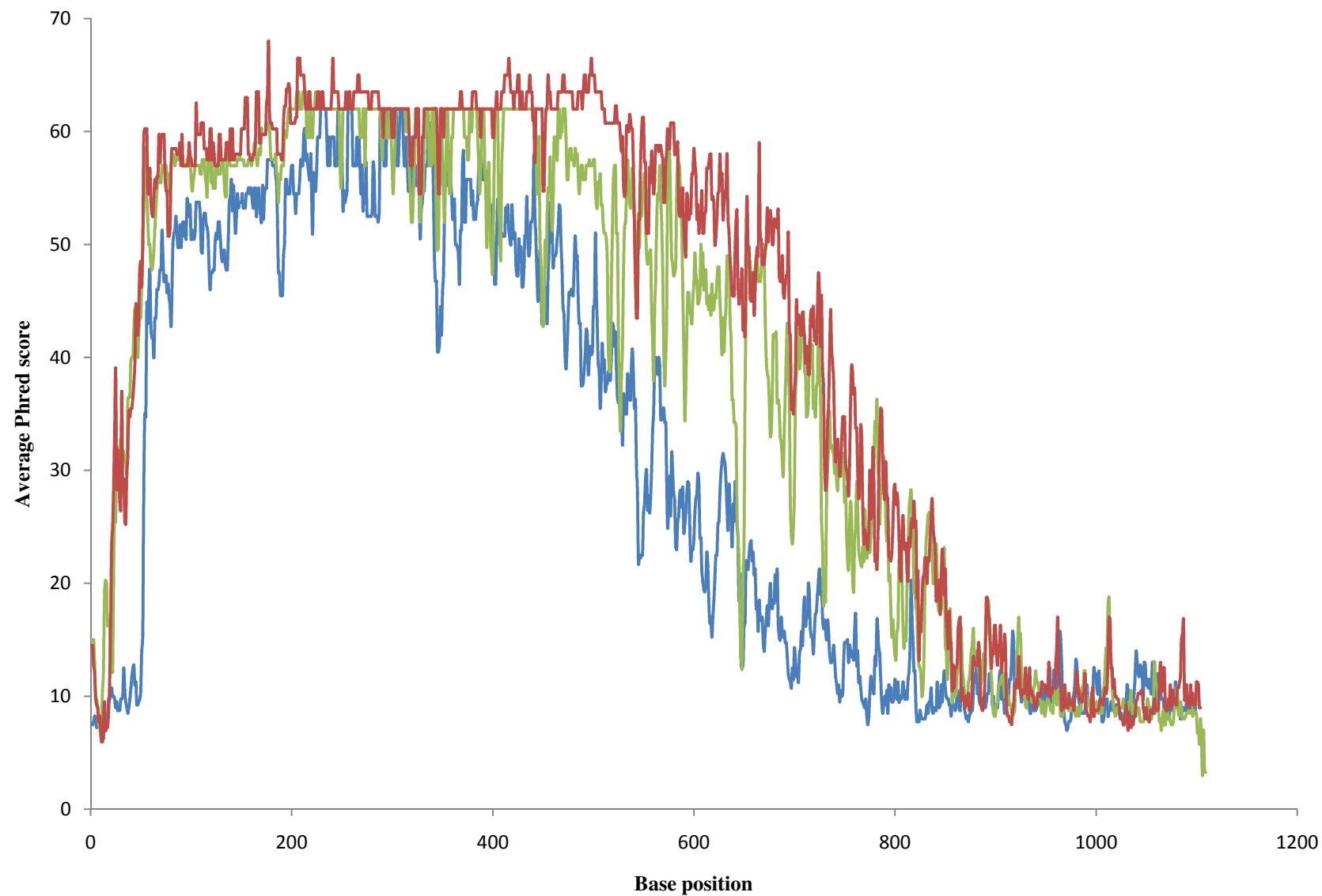

Supplement: Figure S5 — Average Phred score (using CT primer) versus base position. IMAC purified (red) and QIAquick purified (green) samples have better quality scores than unpurified (blue) samples. (0.36 MB PDF) [file pone.0014512.s005.pdf]
