# Supplementary material for: Removal of PCR Error Products and Unincorporated Primers by Metal-Chelate Affinity Chromatography
Source: PLoS One. 2011 Jan 14;6(1):e14512. doi: 10.1371/journal.pone.0014512 (PMC3021510; doi:10.1371/journal.pone.0014512)

IMAC purified1

Length: 1087
October 29, 2010 3:13:07 PM CDT
Page 1 of 1

q>=20: 959
q>=30: 800
q>=40: 680

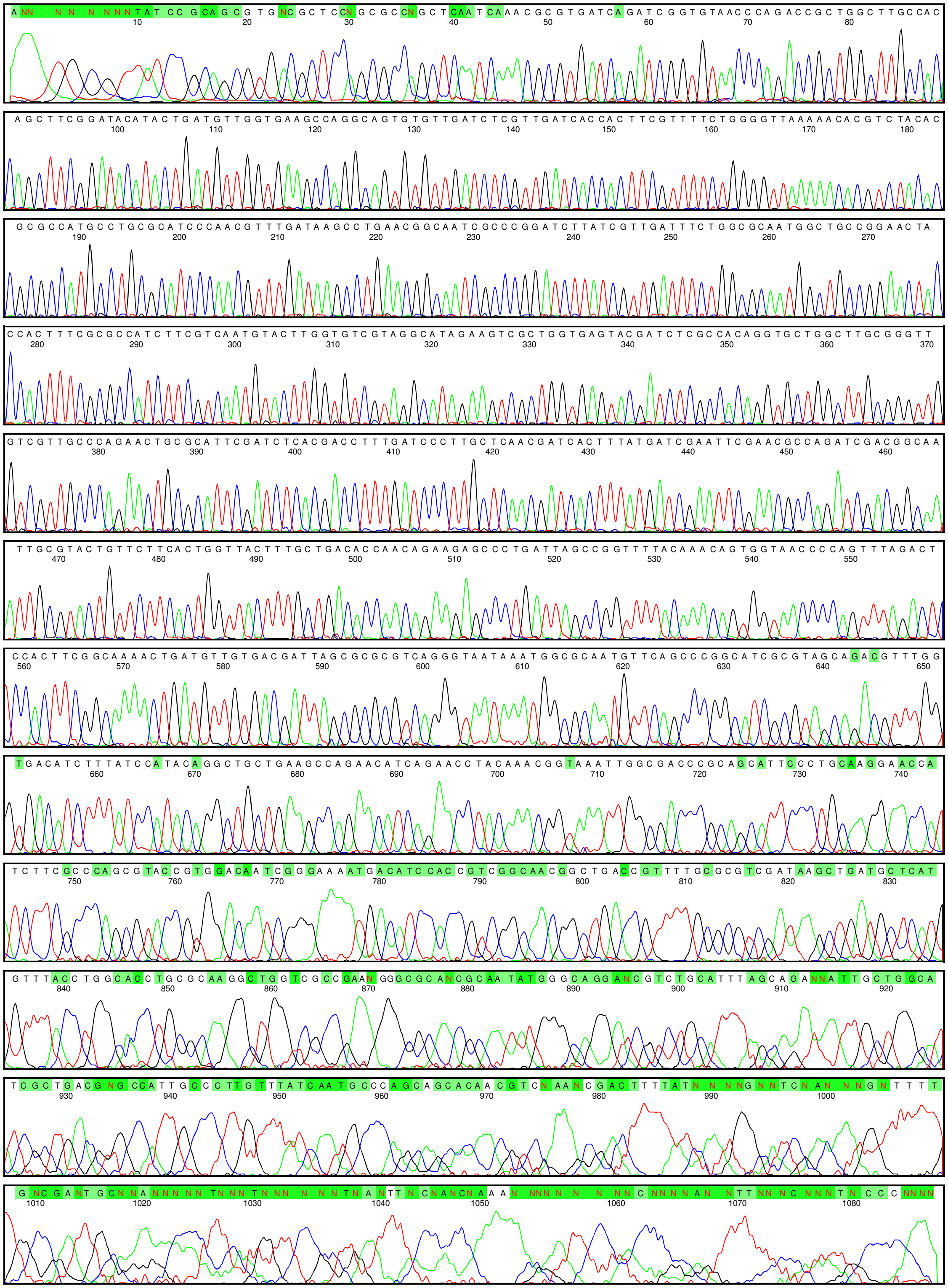

CodonCode Aligner

IMAC purified2

Length: 1092
October 29, 2010 3:13:08 PM CDT
Page 1 of 1

q>=20: 944
q>=30: 776
q>=40: 656

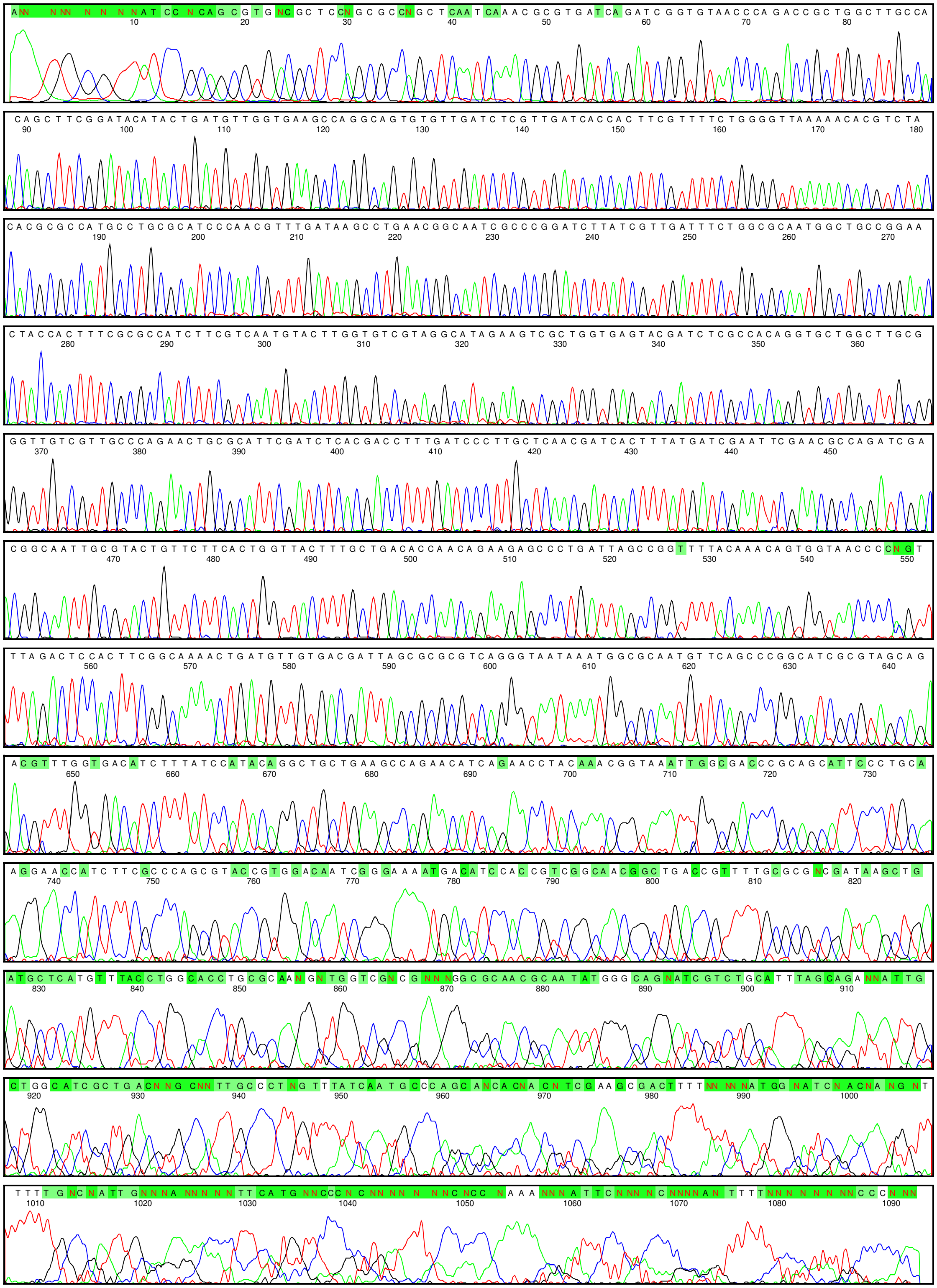

IMAC purified3

Length: 1089
October 29, 2010 3:13:10 PM CDT
Page 1 of 1

q>=20: 980
q>=30: 834
q>=40: 718

CodonCode Aligner

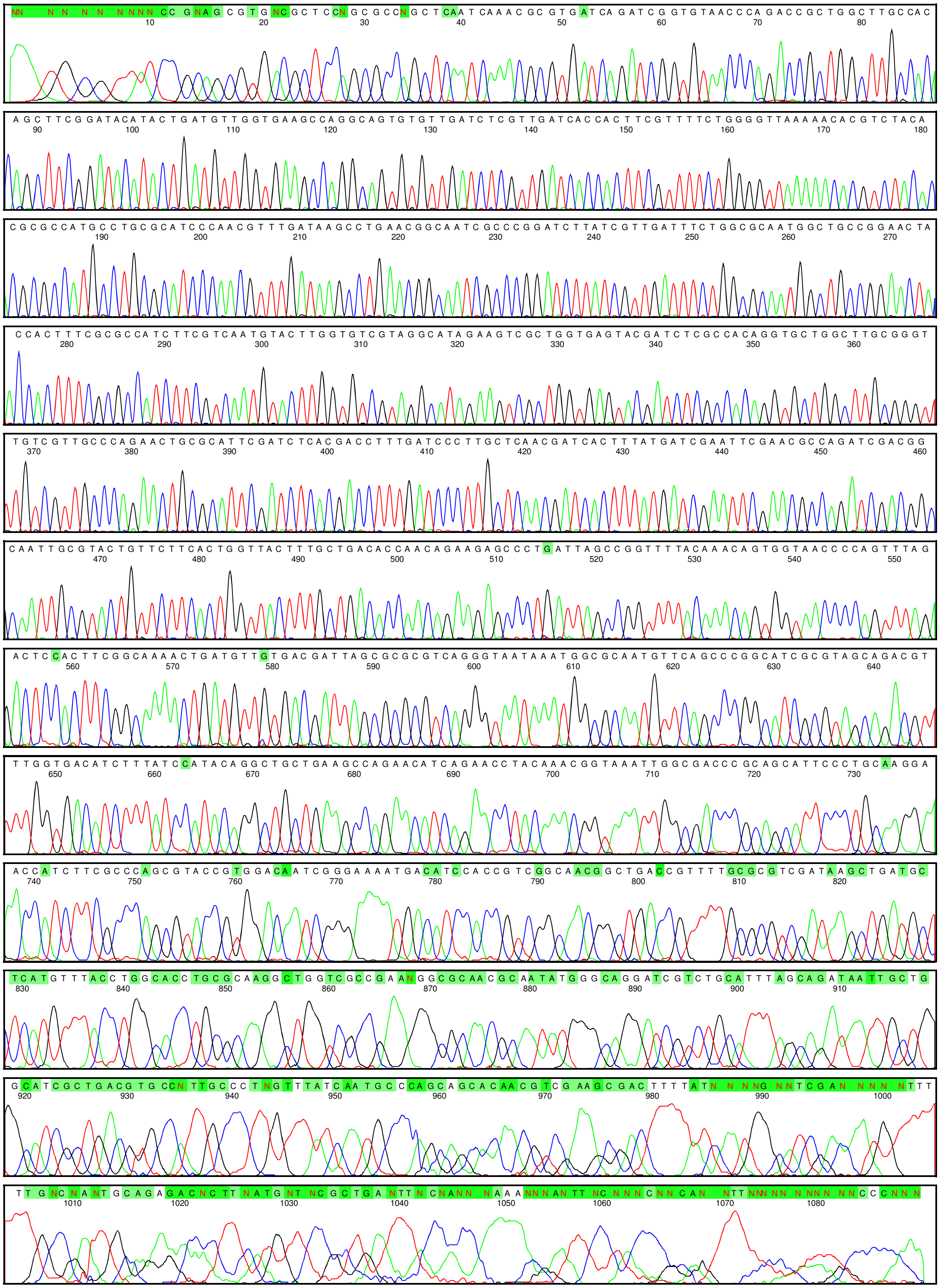

IMAC purified4

Length: 1092
October 29, 2010 3:13:11 PM CDT

q>=20: 975
q>=30: 780
q>=40: 658

CodonCode Aligner

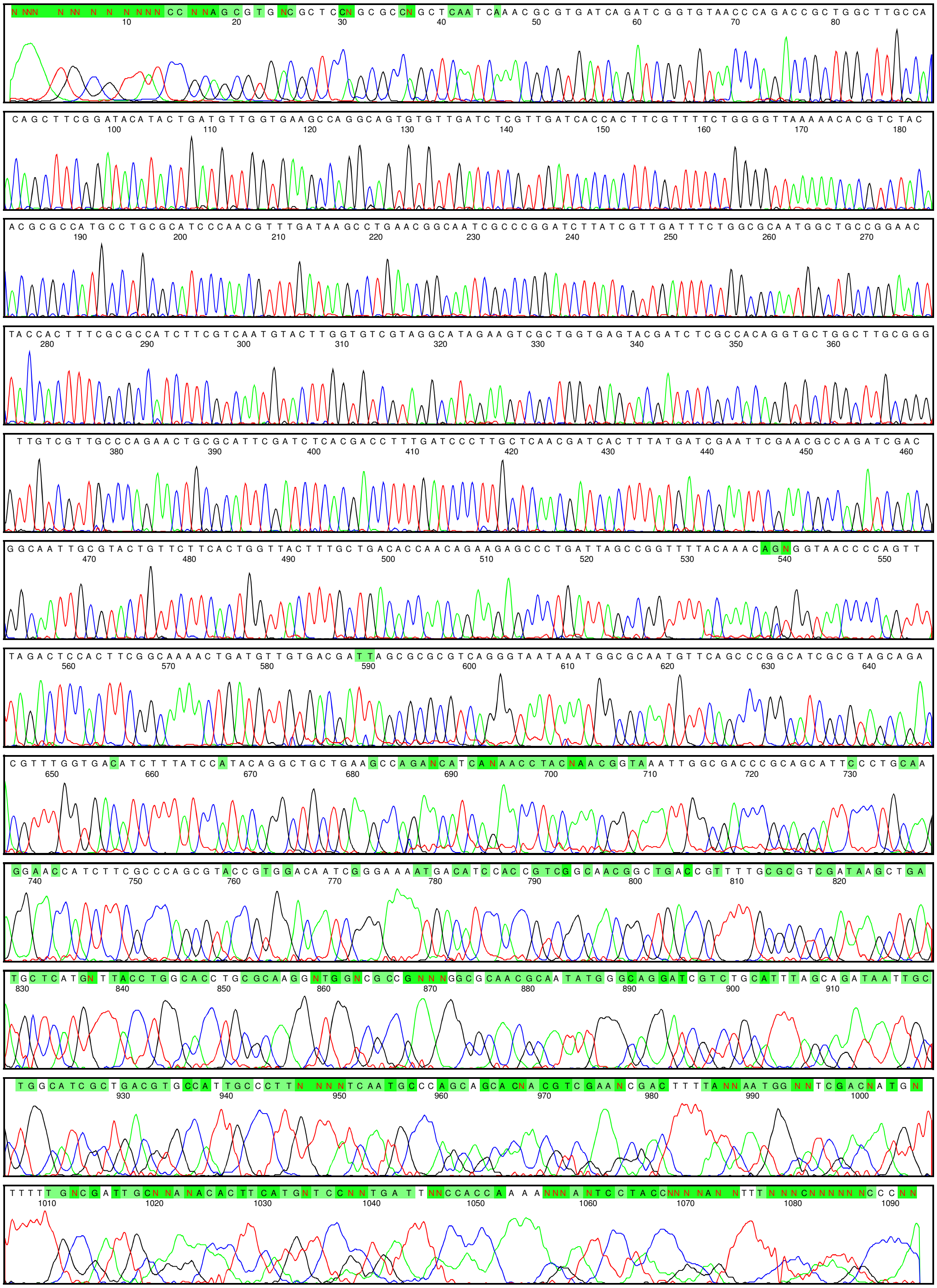

QIAquick purified1

Length: 1097
October 29, 2010 3:13:13 PM CDT
Page 1 of 1

q>=20: 895
q>=30: 725
q>=40: 594

CodonCode Aligner

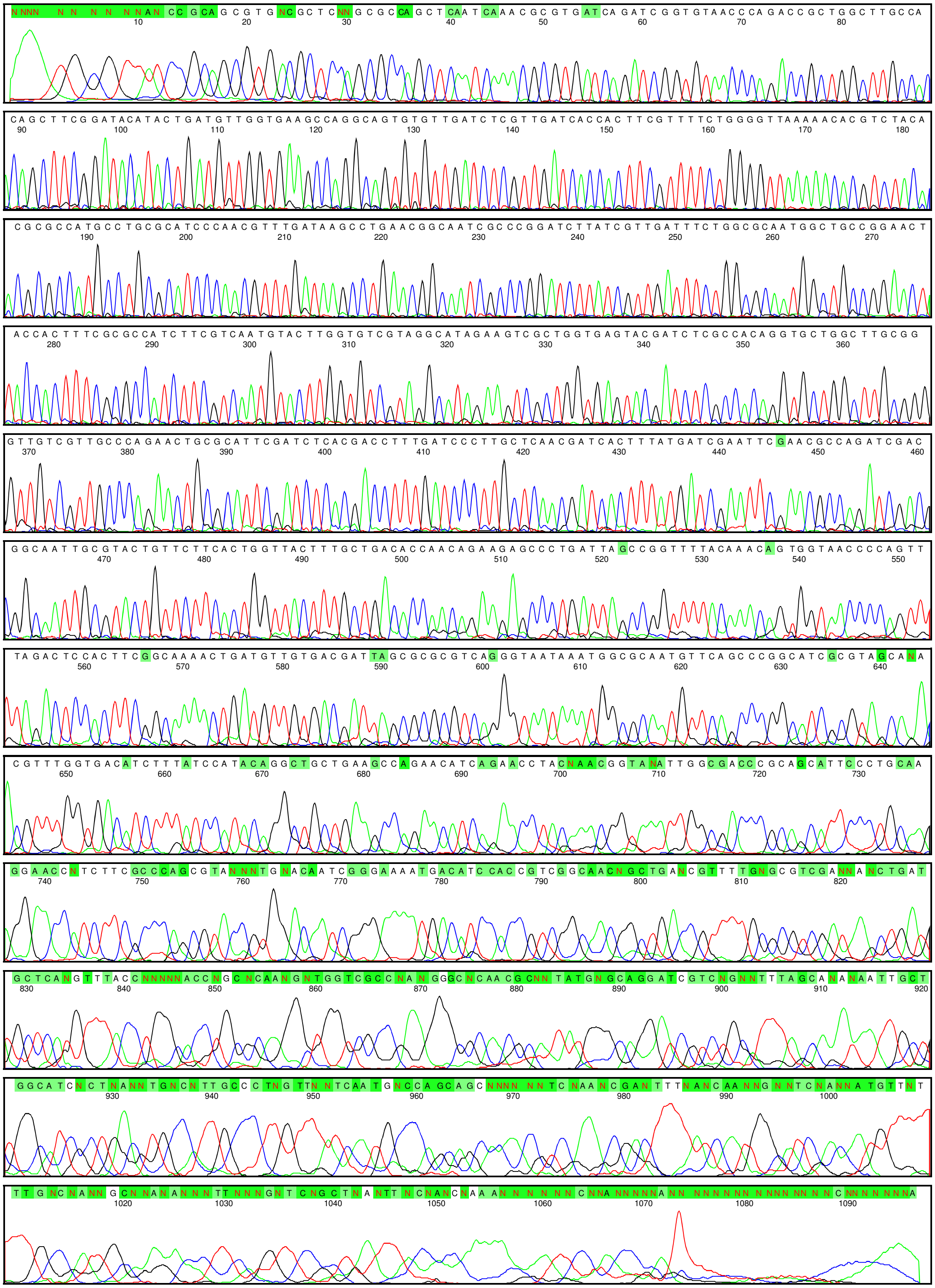

QIAquick purified2

Length: 1075
October 29, 2010 3:13:14 PM CDT

q>=20: 933
q>=30: 773
q>=40: 656

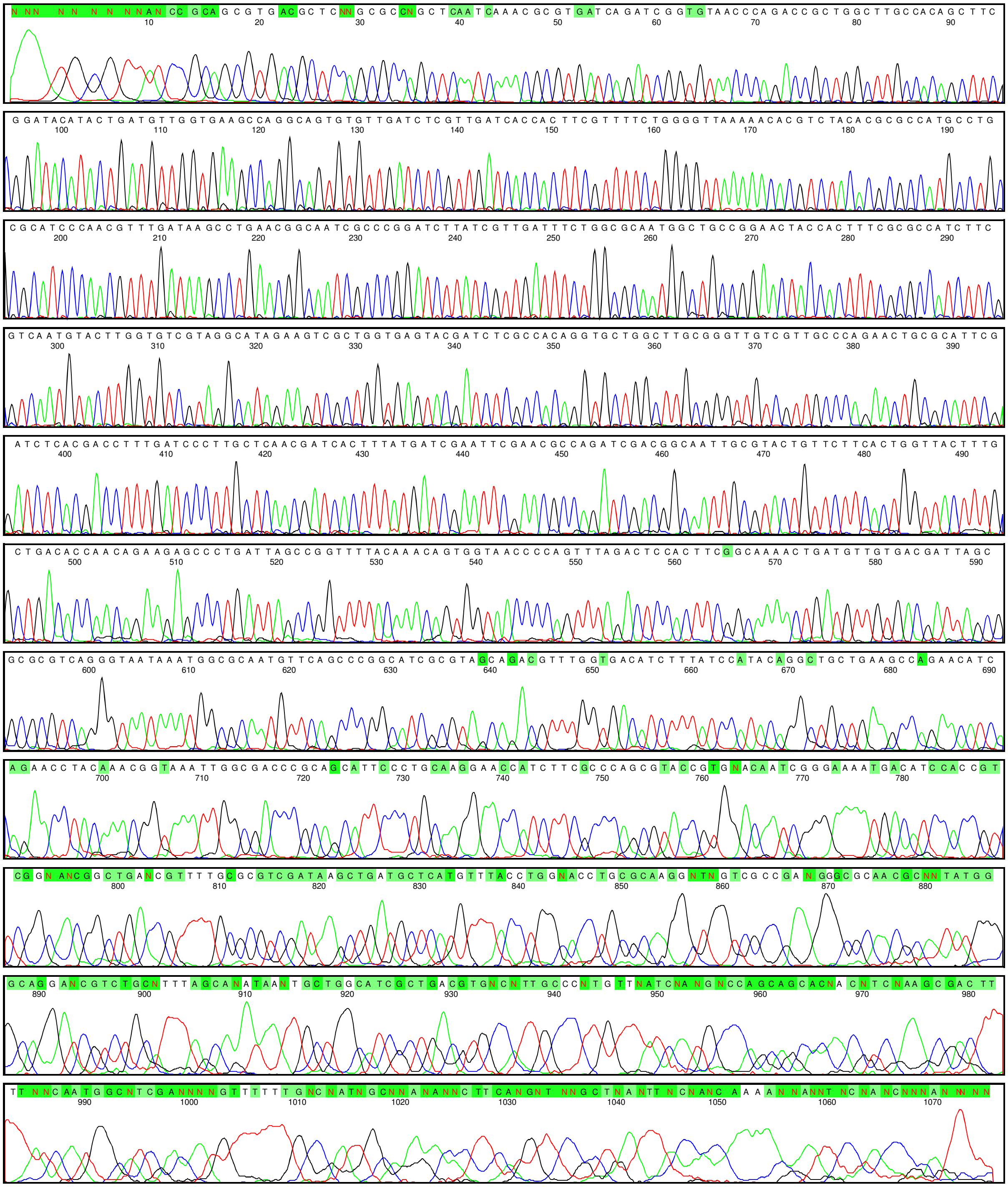

QIAquick purified3

Length: 1079
October 29, 2010 3:13:16 PM CDT

q>=20: 936
q>=30: 772
q>=40: 647

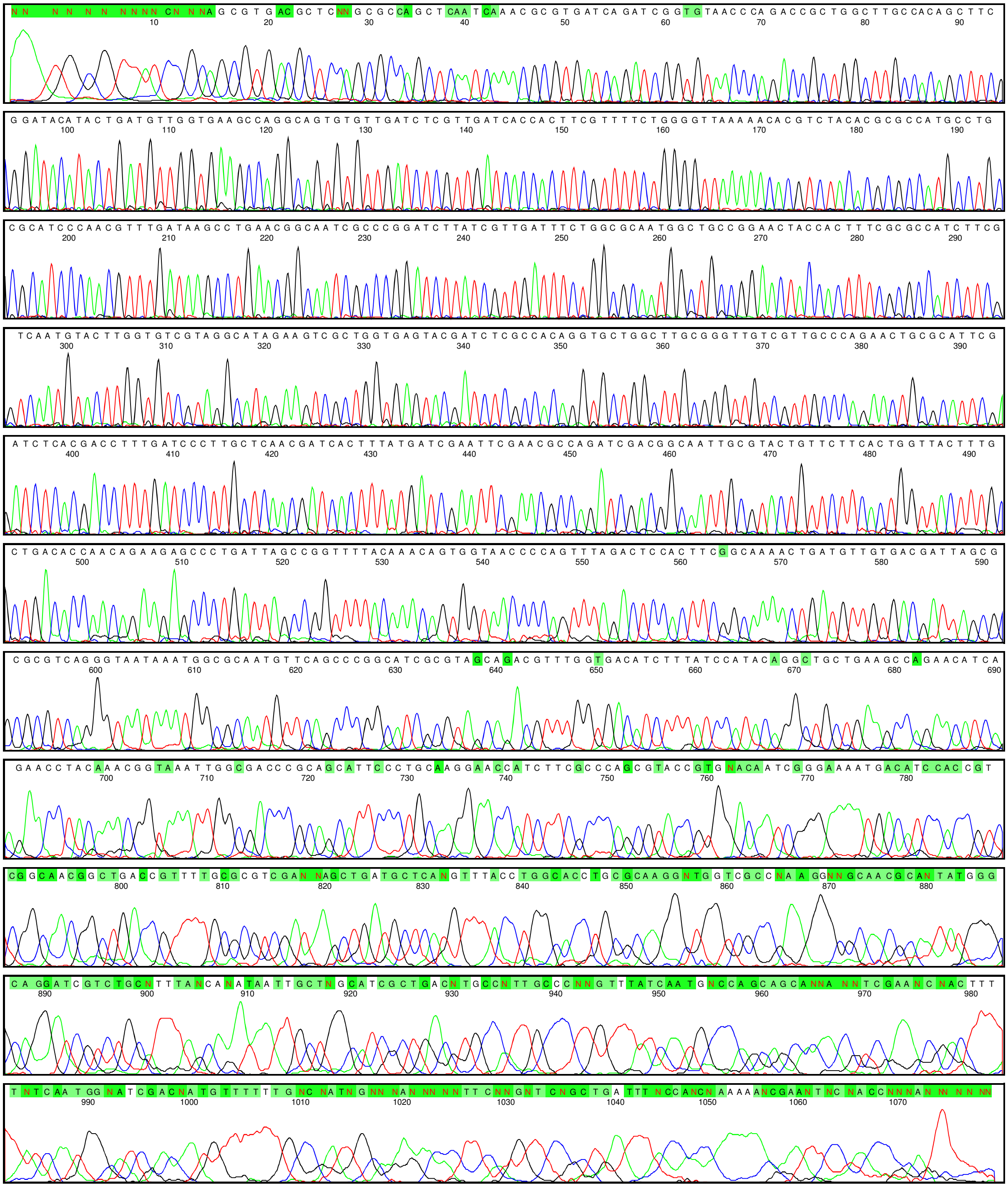

QIAquick purified4

Length: 1091
October 29, 2010 3:13:18 PM CDT
Page 1 of 1

q>=20: 911
q>=30: 776
q>=40: 647

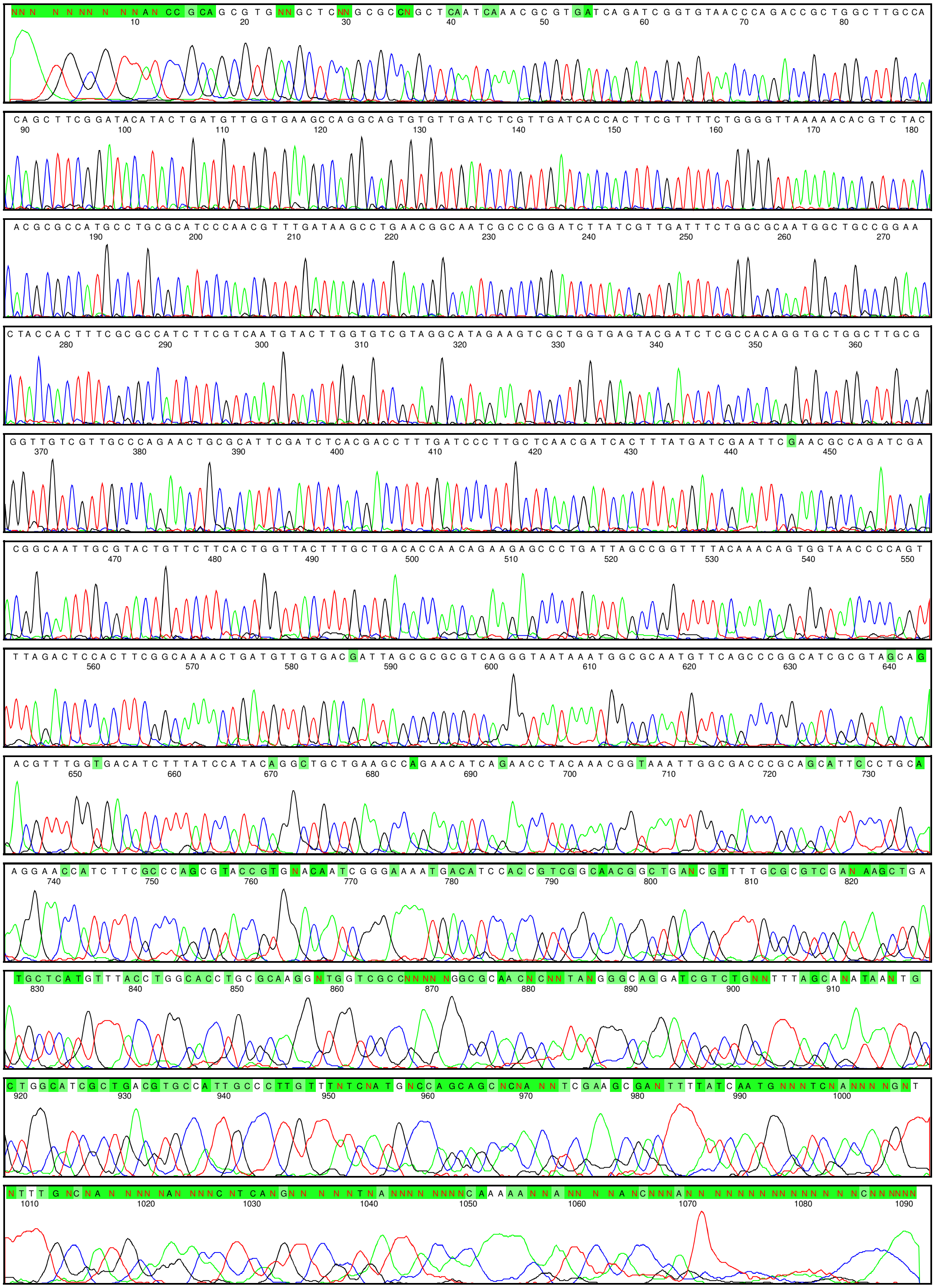

Unpurified1

Length: 1117
October 29, 2010 3:13:19 PM CDT
Page 1 of 1

q>=20: 698
q>=30: 520
q>=40: 351

CodonCode Aligner

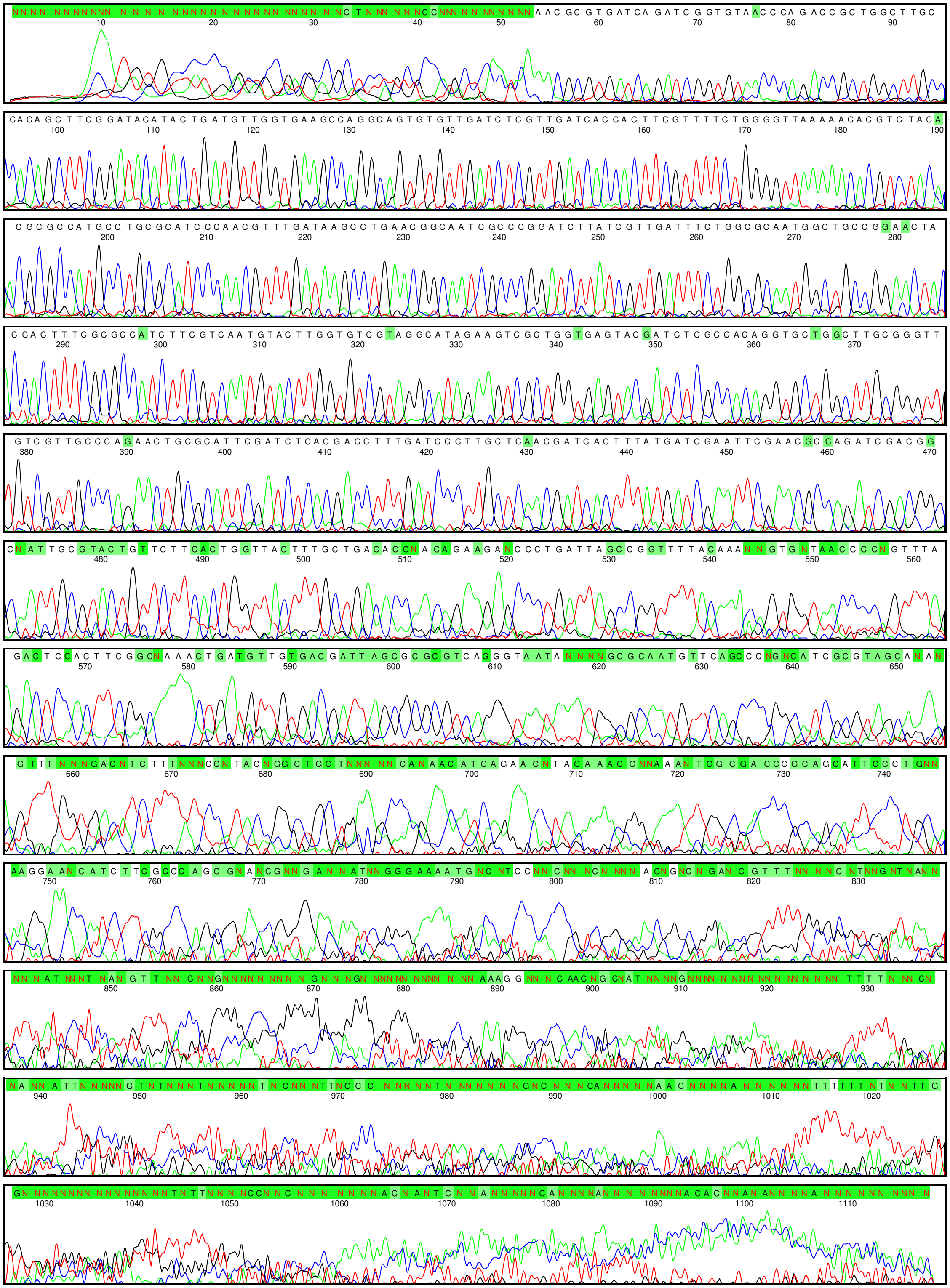

Unpurified2

Length: 1100
October 29, 2010 3:13:21 PM CDT
Page 1 of 1

q>=20: 684
q>=30: 526
q>=40: 346

CodonCode Aligner

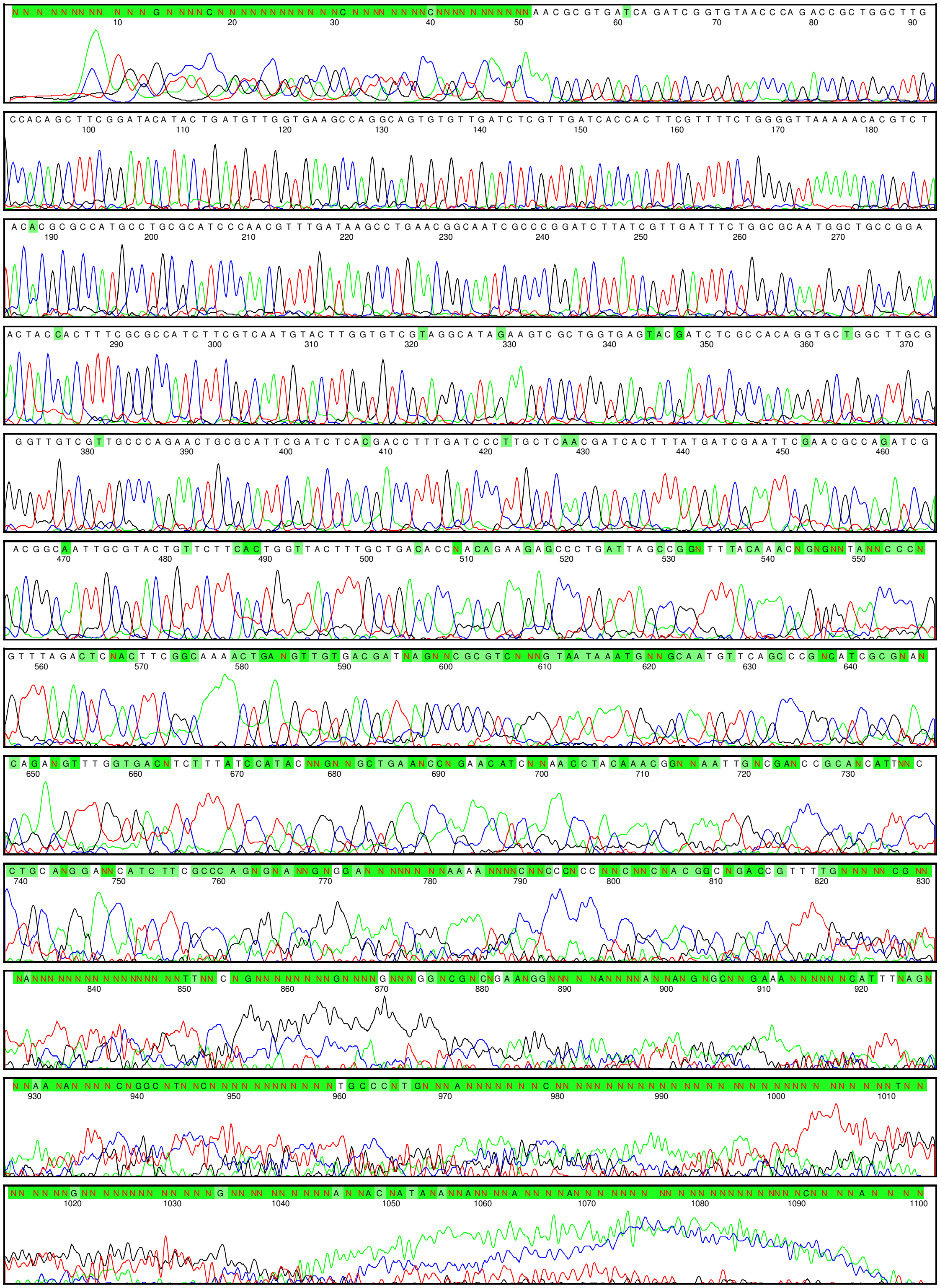

Unpurified3

Length: 1078

October 29, 2010 3:13:23 PM CDT

Page 1 of 1

q>=20: 820

q>=30: 663

q>=40: 551

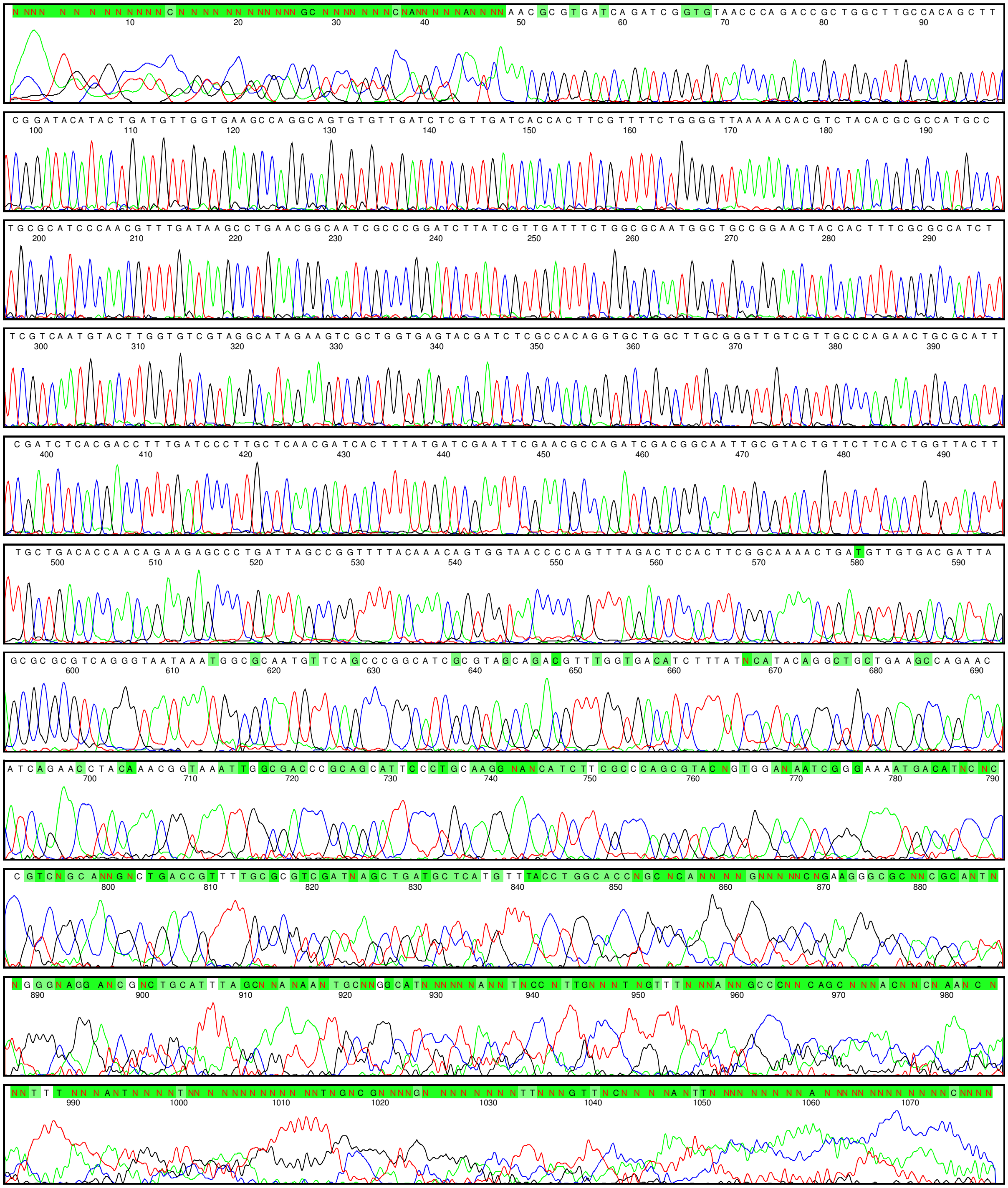

CodonCode Aligner

Unpurified4

Length: 1103
October 29, 2010 3:13:25 PM CDT
Page 1 of 1

q>=20: 757
q>=30: 610
q>=40: 469

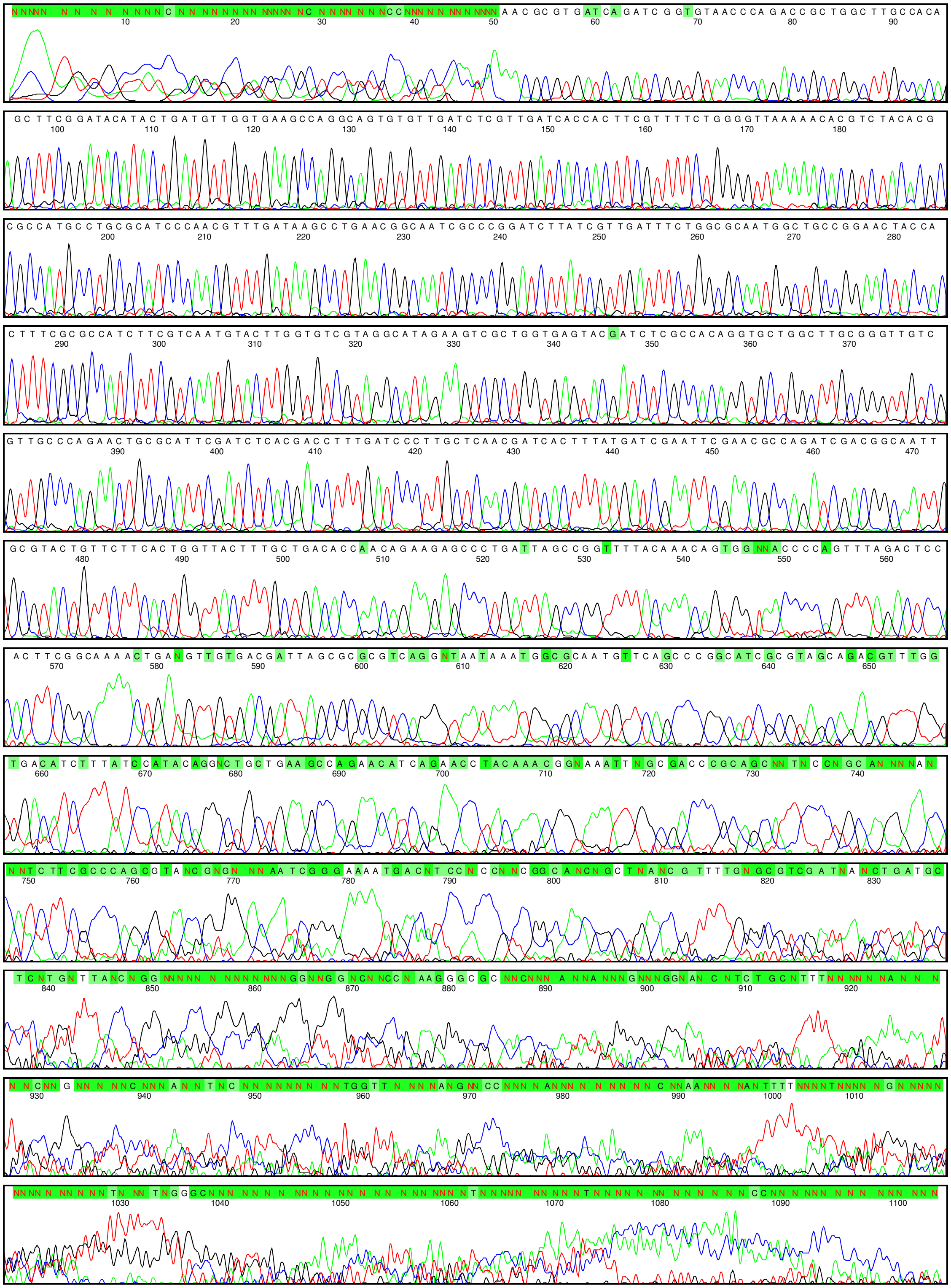

Supplement: Figure S2 — Raw sequence trace using CT primer. (5.44 MB PDF) [file pone.0014512.s002.pdf]
