# Supplementary material for: Removal of PCR Error Products and Unincorporated Primers by Metal-Chelate Affinity Chromatography
Source: PLoS One. 2011 Jan 14;6(1):e14512. doi: 10.1371/journal.pone.0014512 (PMC3021510; doi:10.1371/journal.pone.0014512)

IMAC purified1

Length: 1097
October 29, 2010 3:14:33 PM CDT

q>=20: 927
q>=30: 772
q>=40: 629

CodonCode Aligner

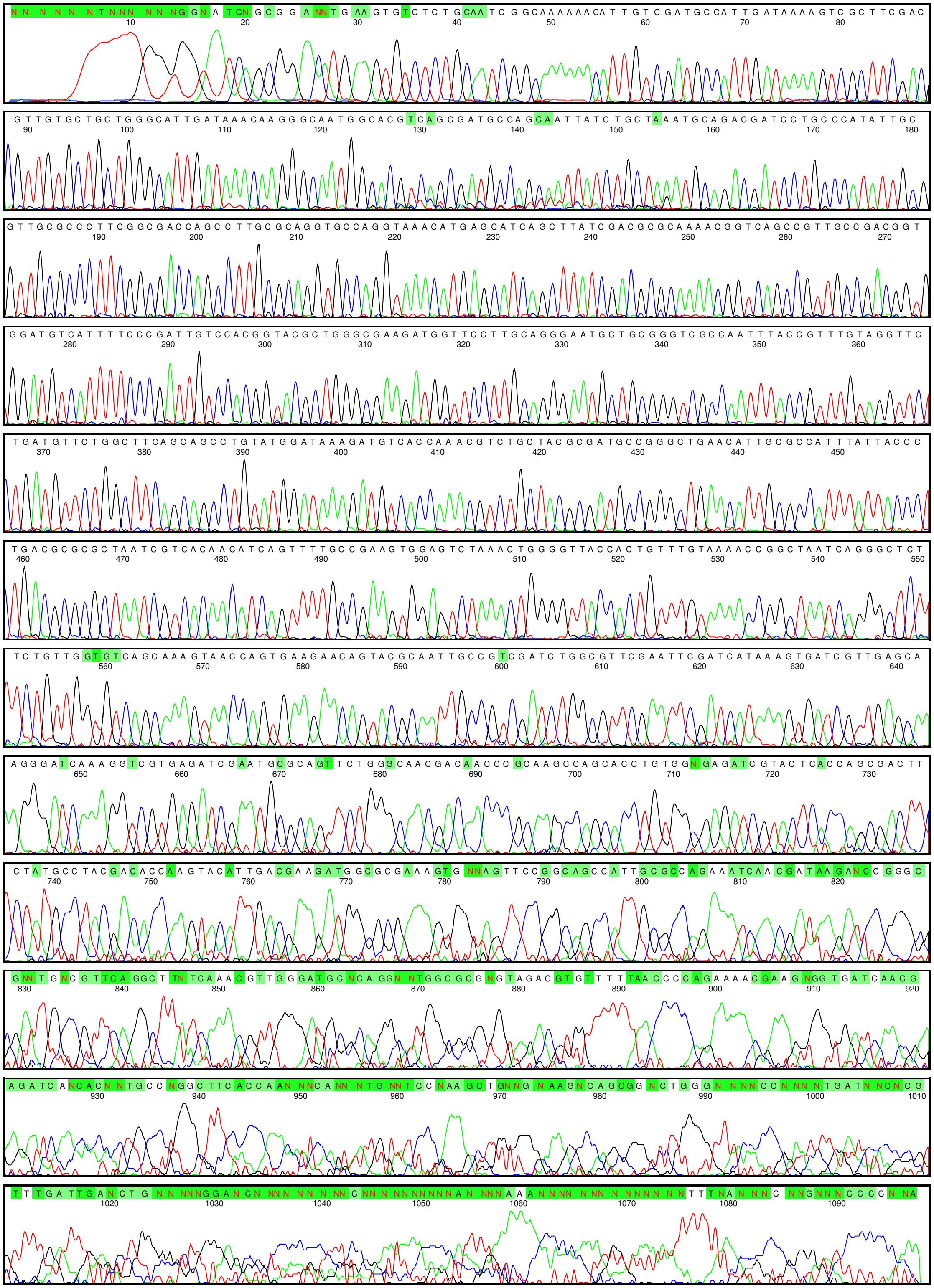

IMAC purified2

CodonCode Aligner

Length: 1096
October 29, 2010 3:14:34 PM CDT
Page 1 of 1

q>=20: 919
q>=30: 764
q>=40: 648

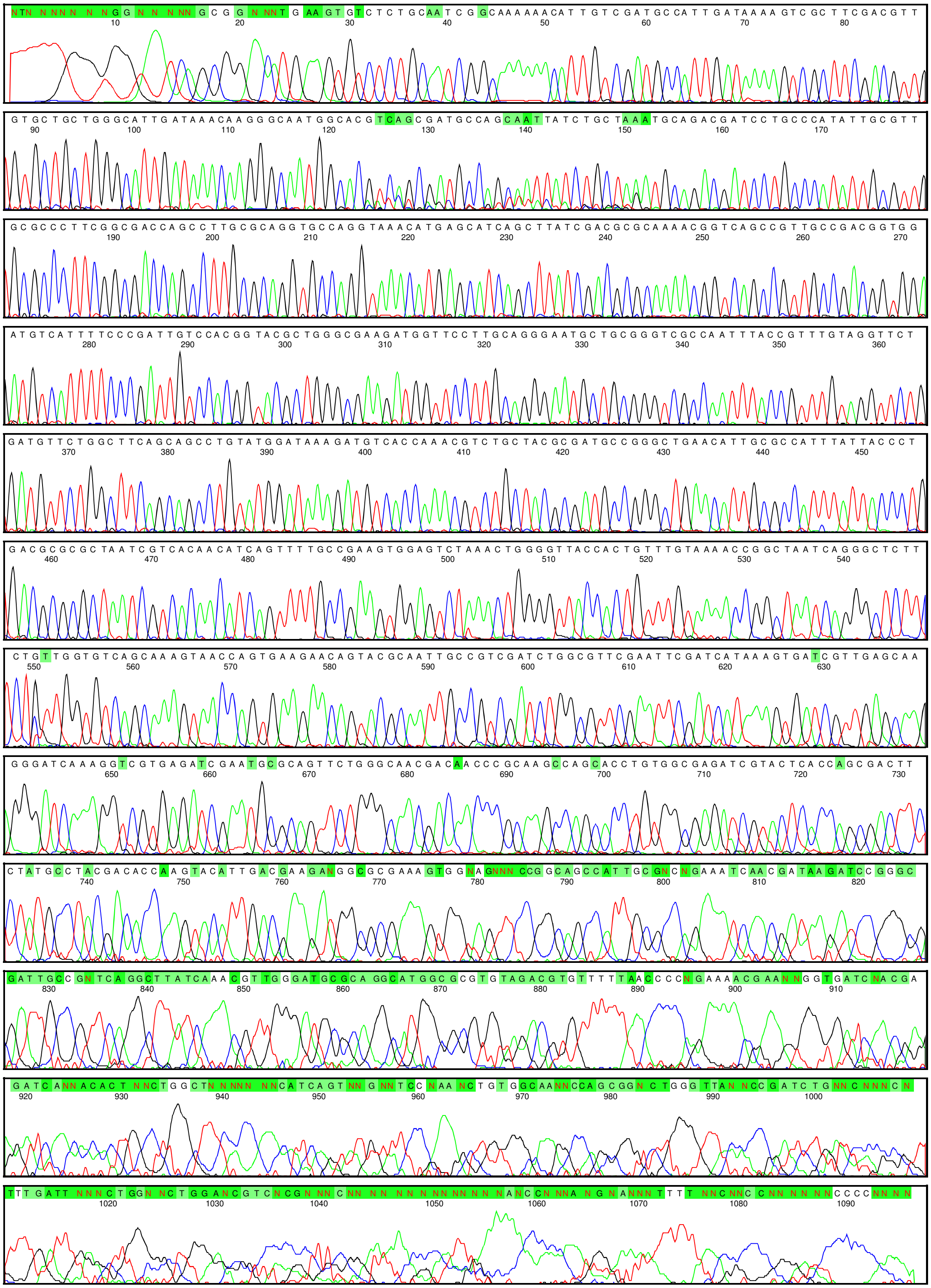

IMAC purified3

Length: 1094
October 29, 2010 3:14:36 PM CDT

q>=20: 934
q>=30: 772
q>=40: 668

CodonCode Aligner

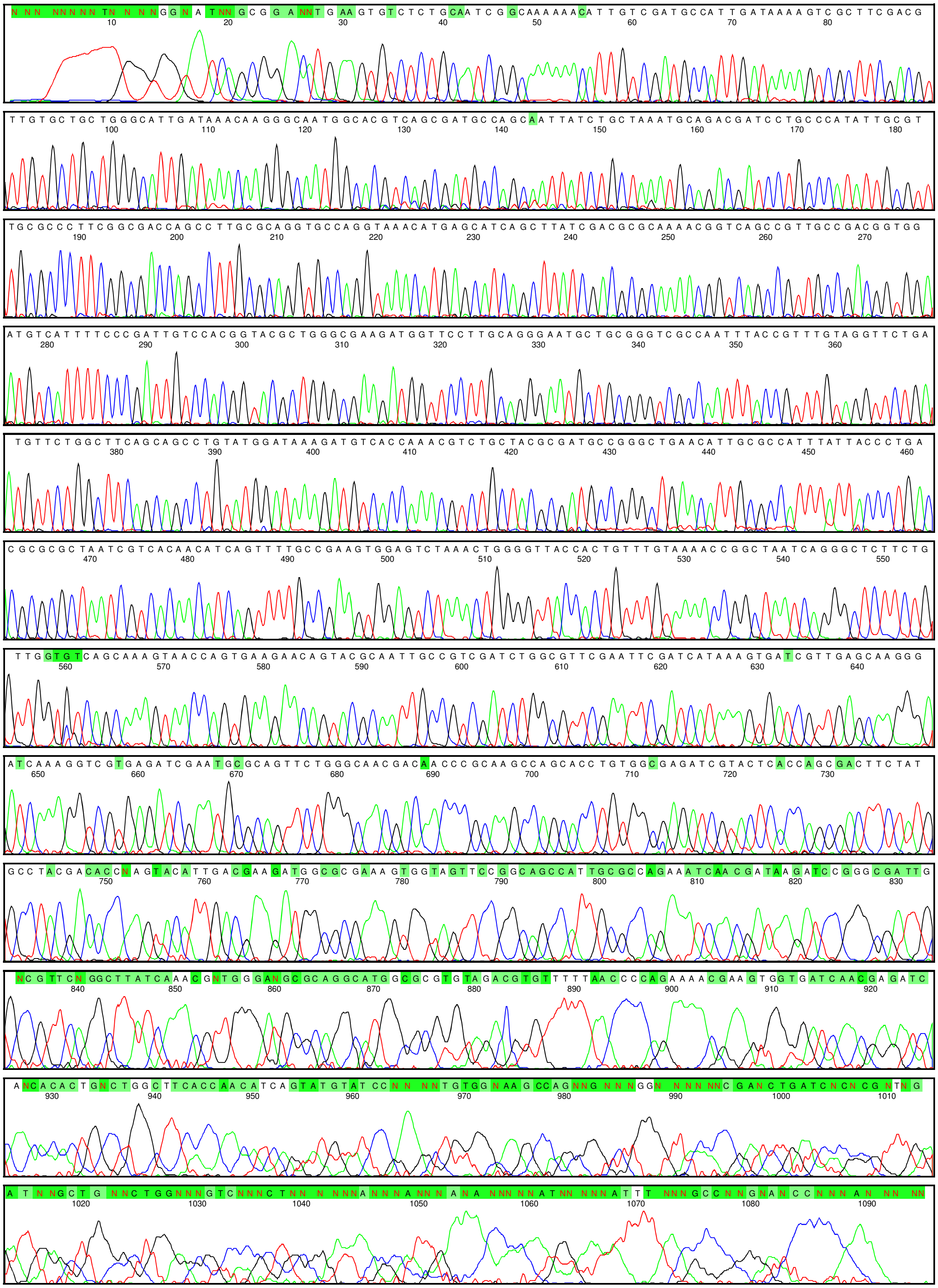

CodonCode Aligner

IMAC purified4

Length: 1094
October 29, 2010 3:14:37 PM CDT
Page 1 of 1

q>=20: 941
q>=30: 780
q>=40: 673

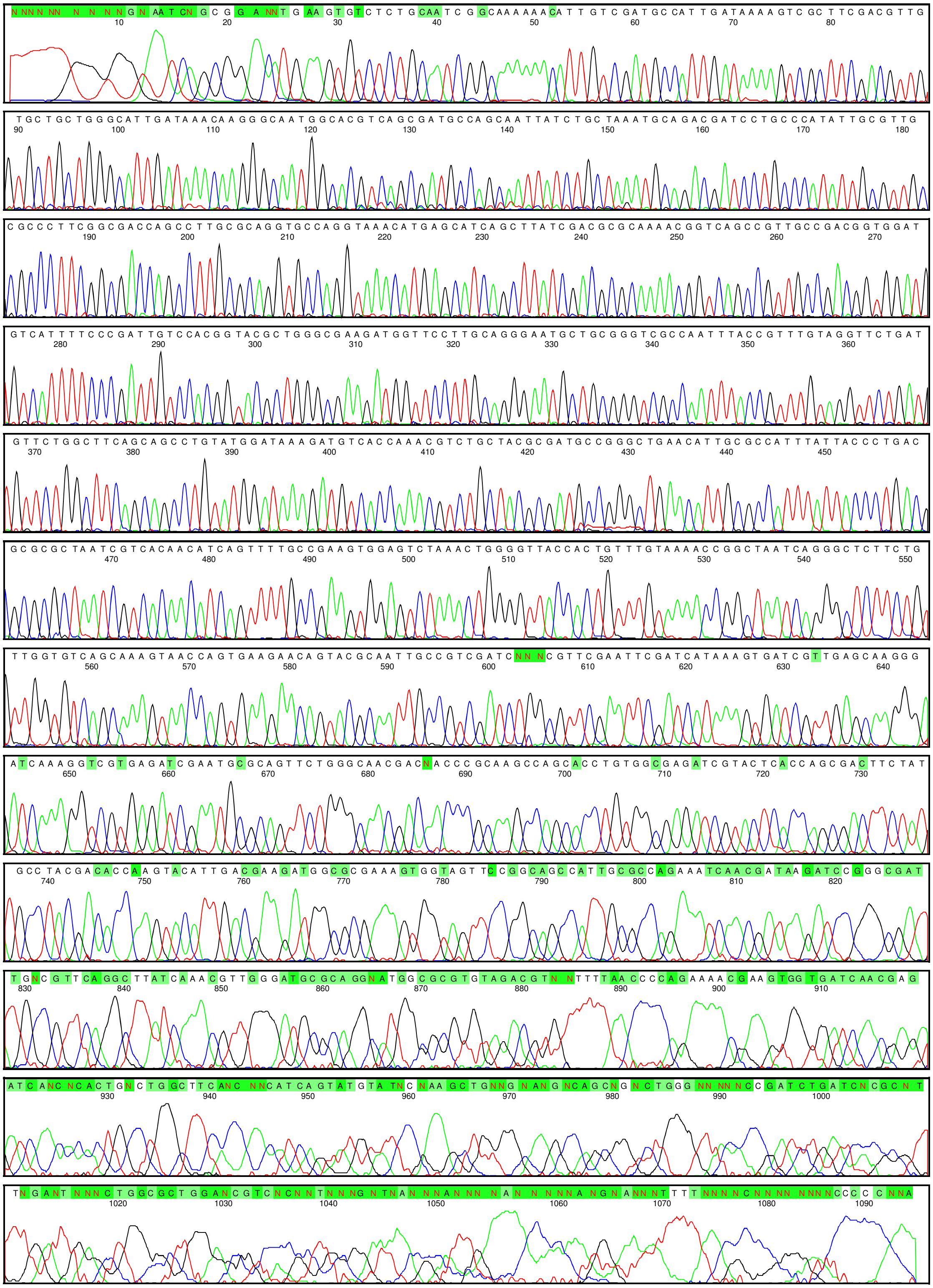

QIAquick purified1

Length: 1103
October 29, 2010 3:14:39 PM CDT
Page 1 of 1

q>=20: 786
q>=30: 598
q>=40: 430

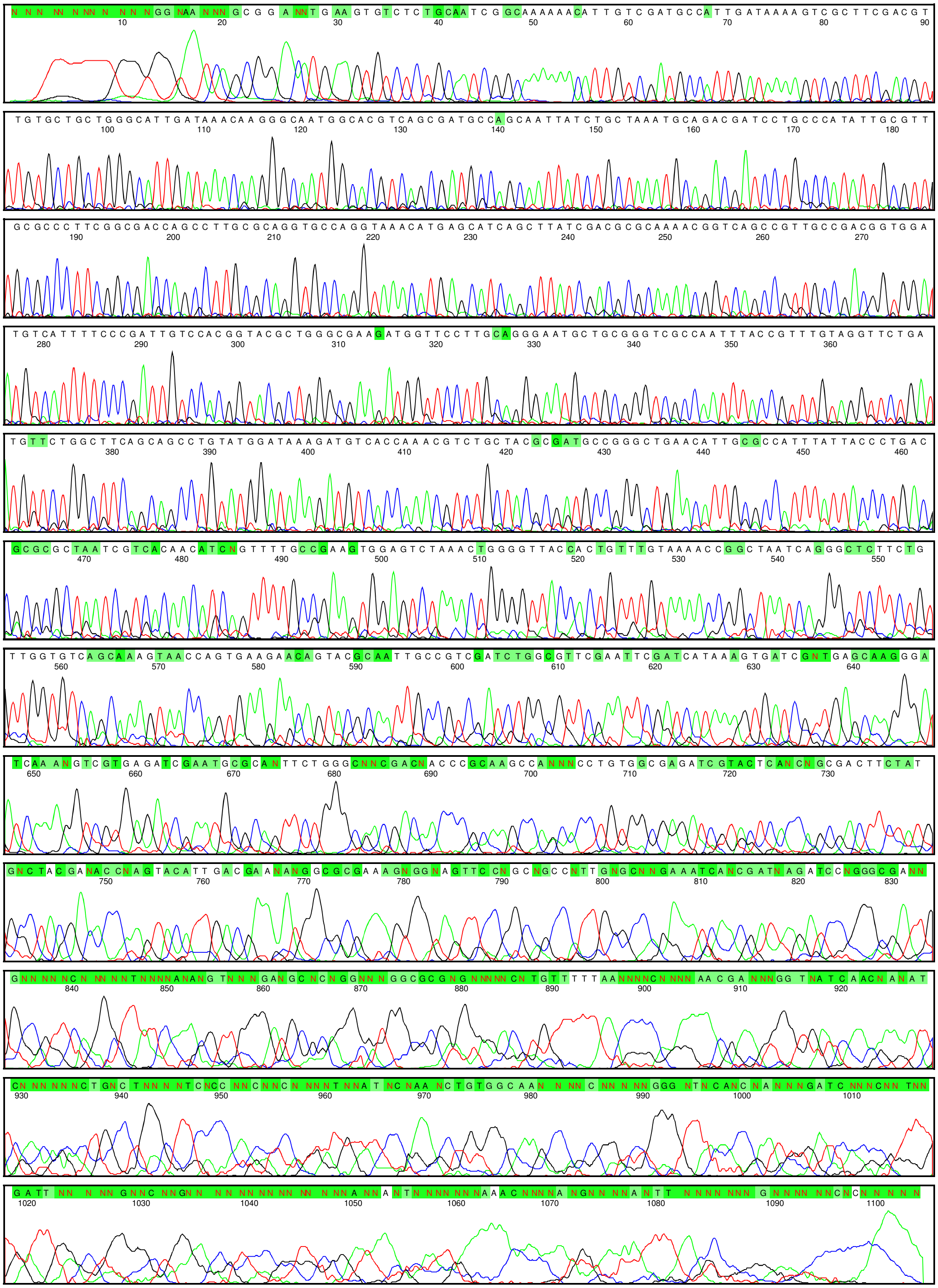

QIAquick purified2

Length: 1098
October 29, 2010 3:14:41 PM CDT
Page 1 of 1

q>=20: 834
q>=30: 628
q>=40: 447

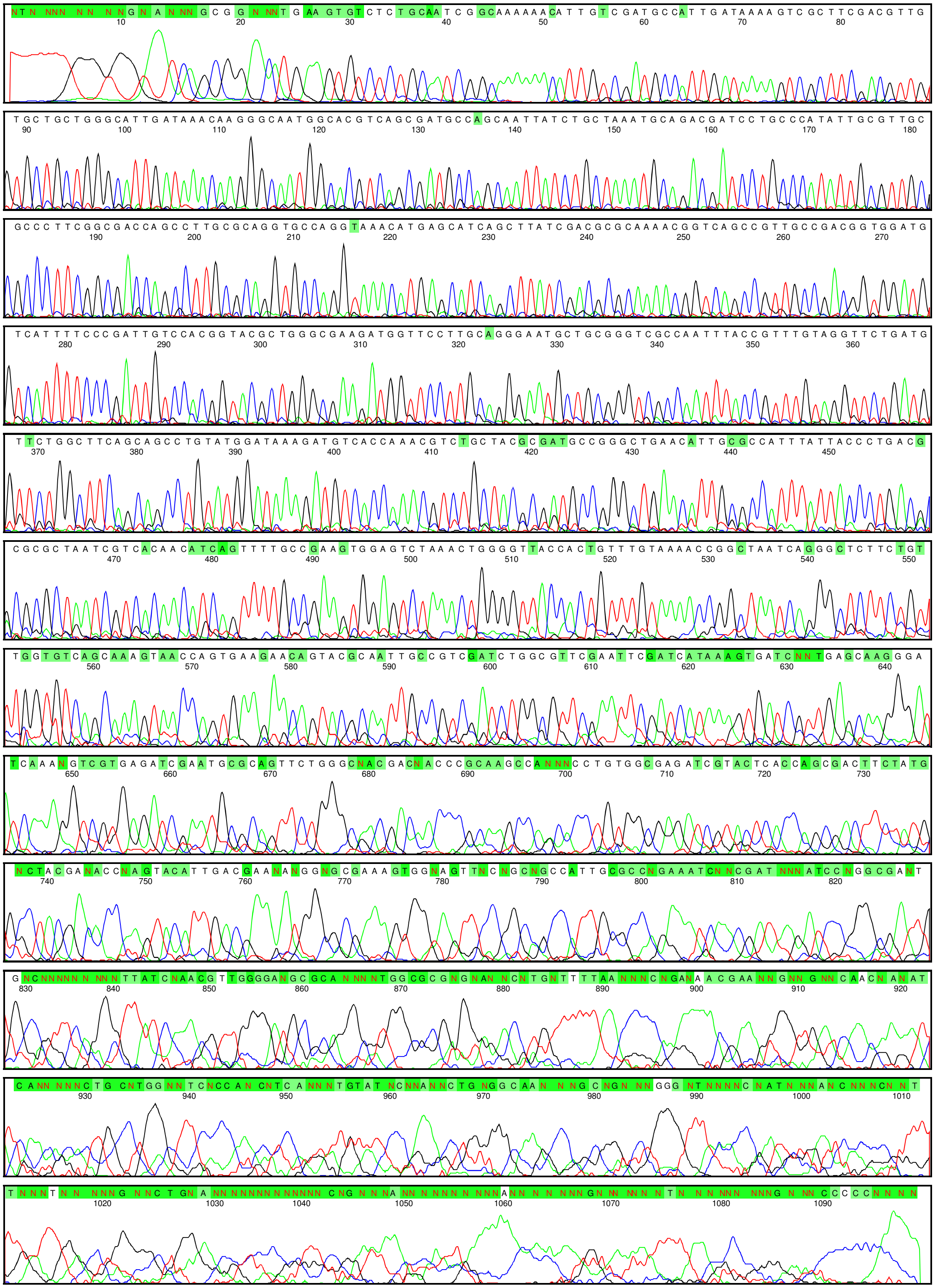

QIAquick purified3

Length: 1106
October 29, 2010 3:14:43 PM CDT
Page 1 of 1

q>=20: 882
q>=30: 705
q>=40: 549

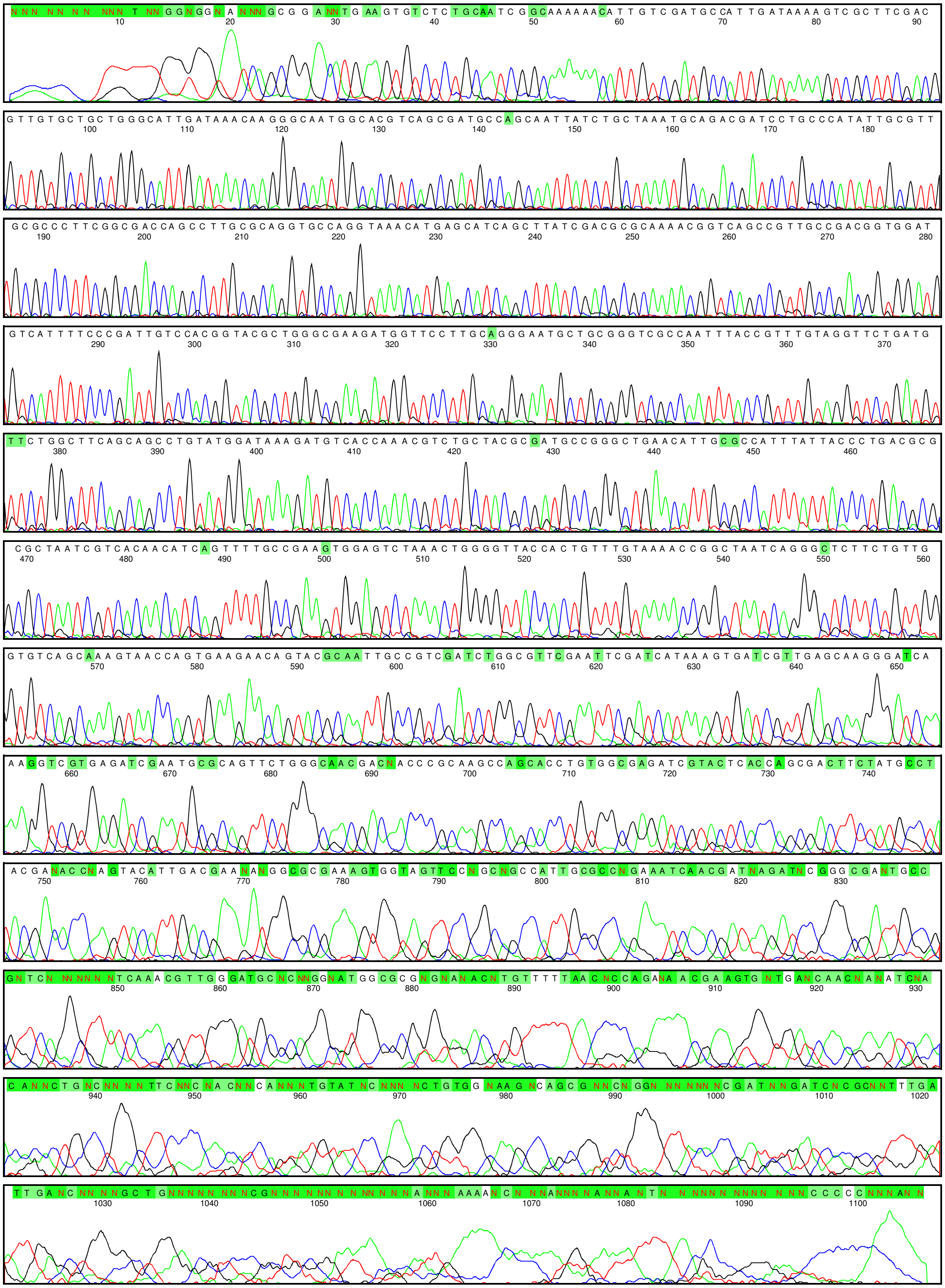

QIAquick purified4

Length: 1100

October 29, 2010 3:14:45 PM CDT

Page 1 of 1

q>=20: 857

q>=30: 673

q>=40: 515

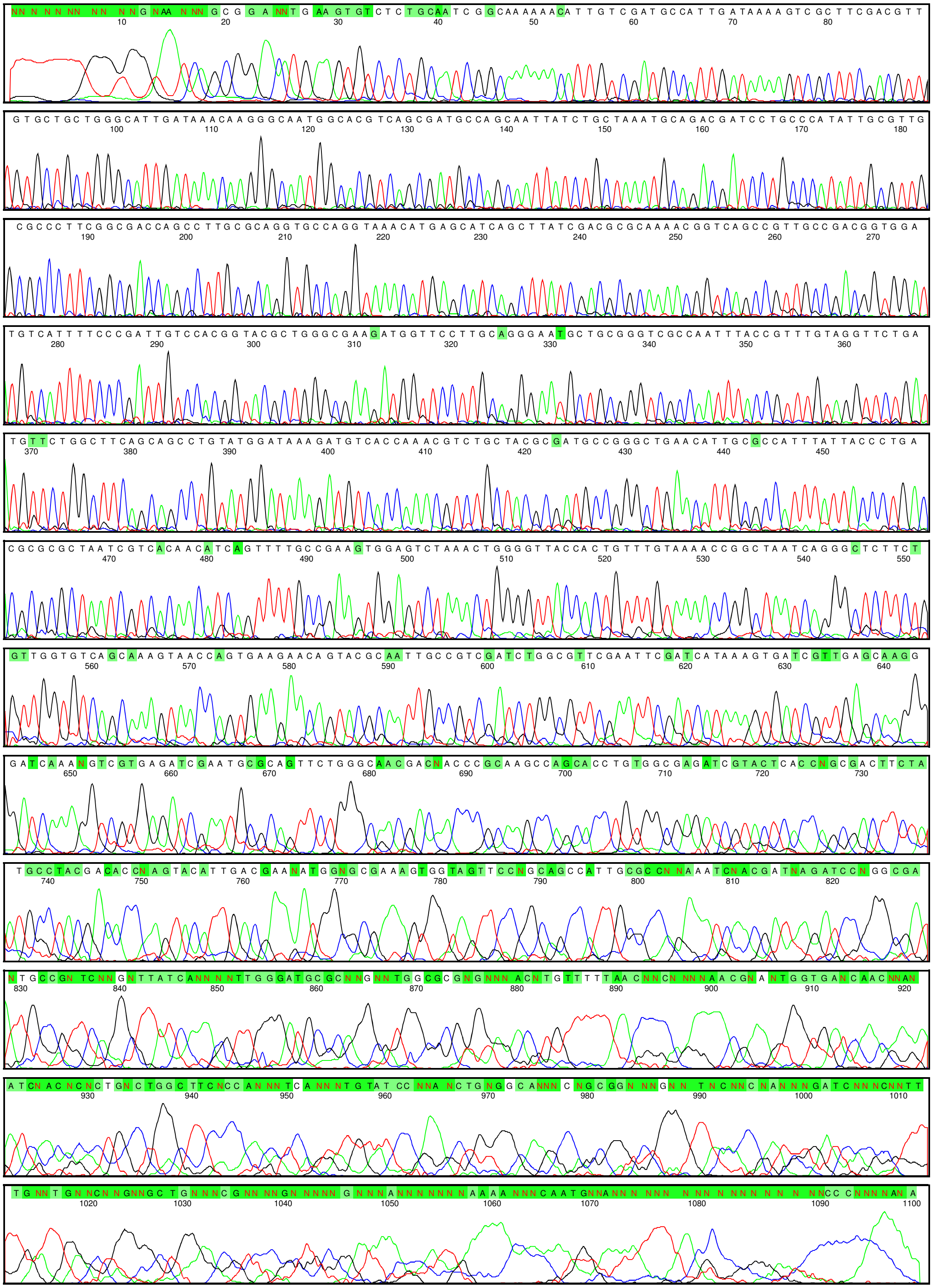

CodonCode Aligner

Unpurified1

Length: 1090
October 29, 2010 3:14:46 PM CDT
Page 1 of 1

q>=20: 755
q>=30: 597
q>=40: 443

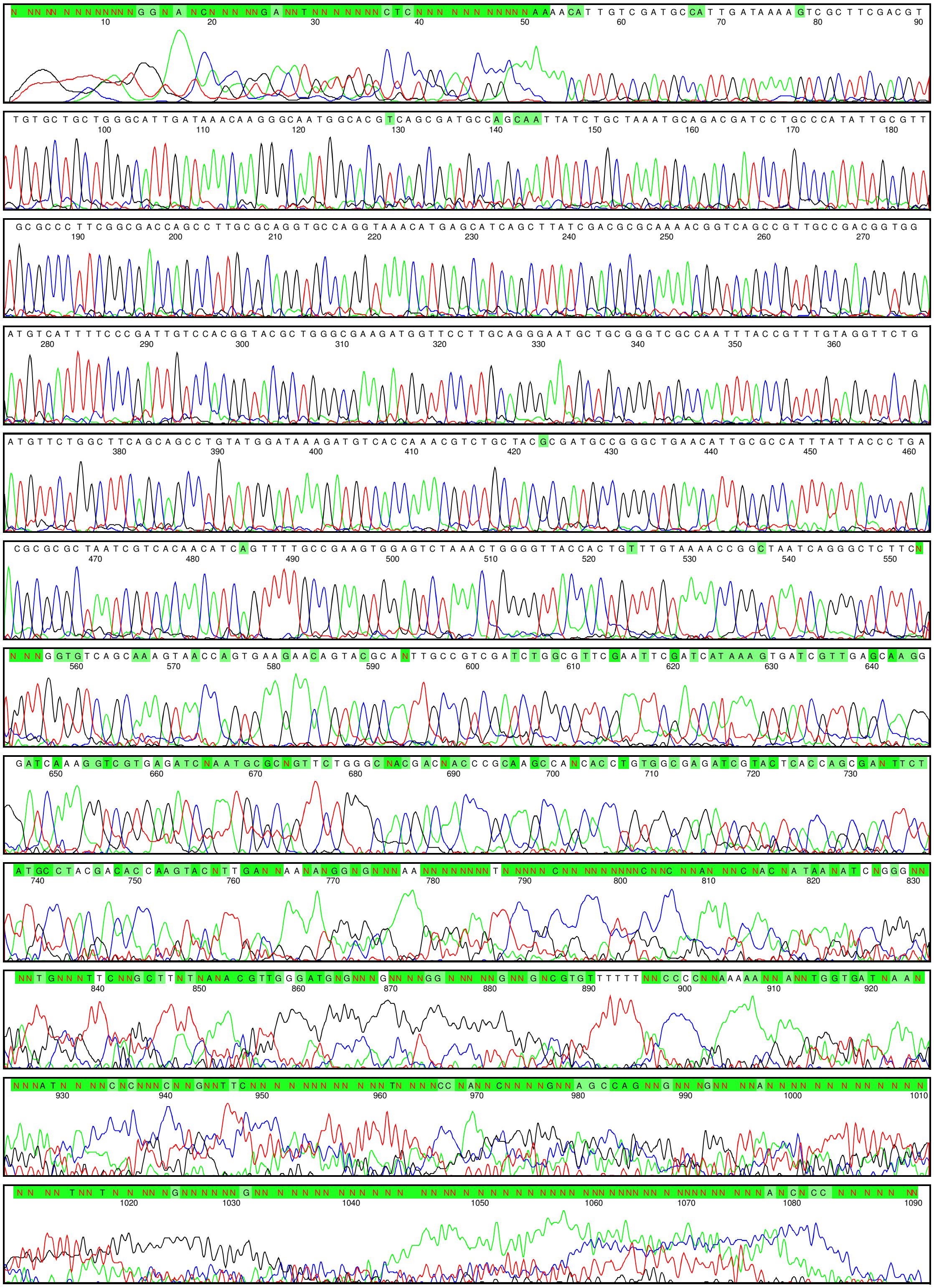

Unpurified2

Length: 1131
October 29, 2010 3:14:48 PM CDT
Page 1 of 1

q>=20: 745
q>=30: 614
q>=40: 442

CodonCode Aligner

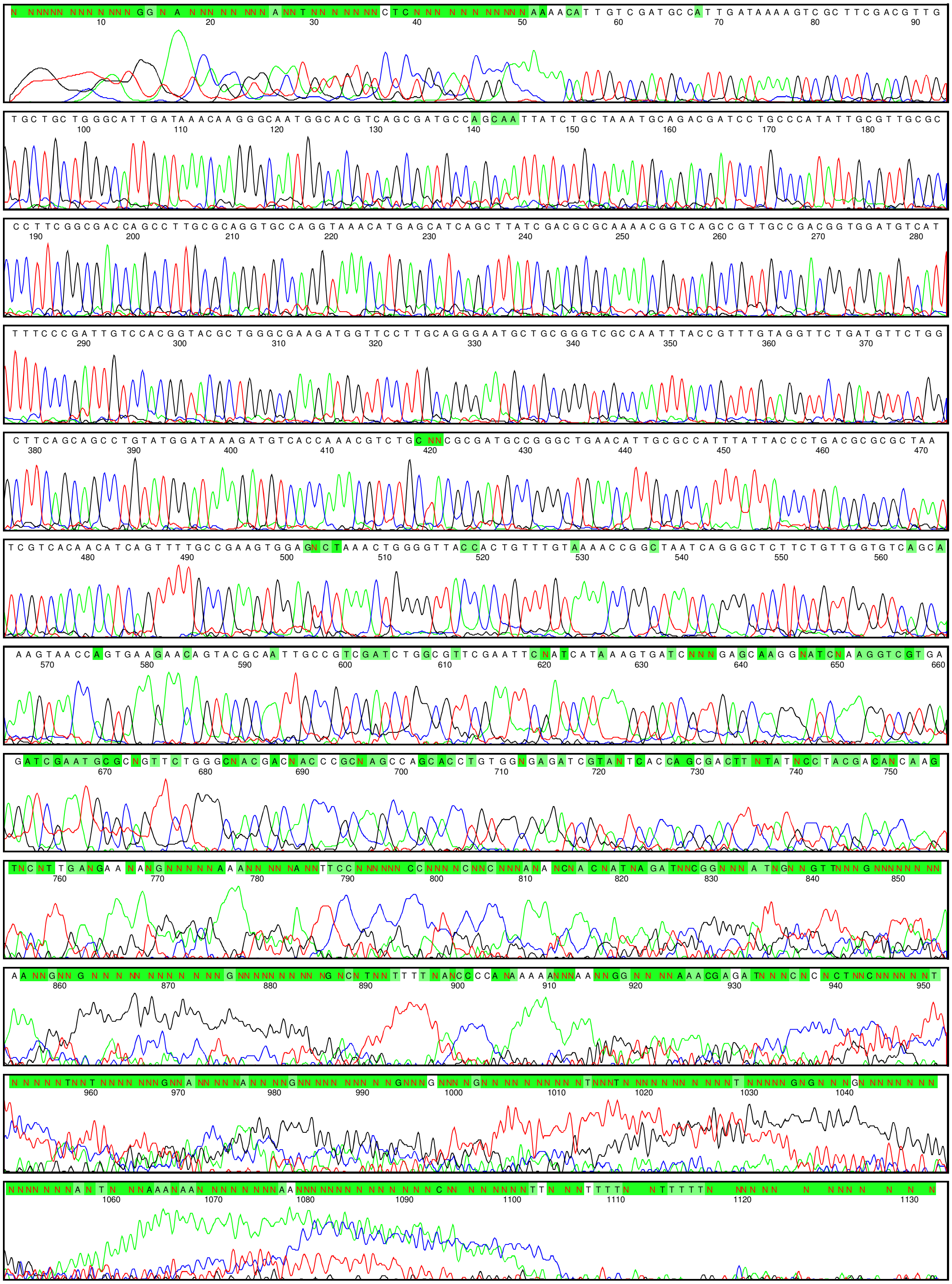

Unpurified3

Length: 1059
October 29, 2010 3:14:49 PM CDT
Page 1 of 1

q>=20: 614
q>=30: 421
q>=40: 178

CodonCode Aligner

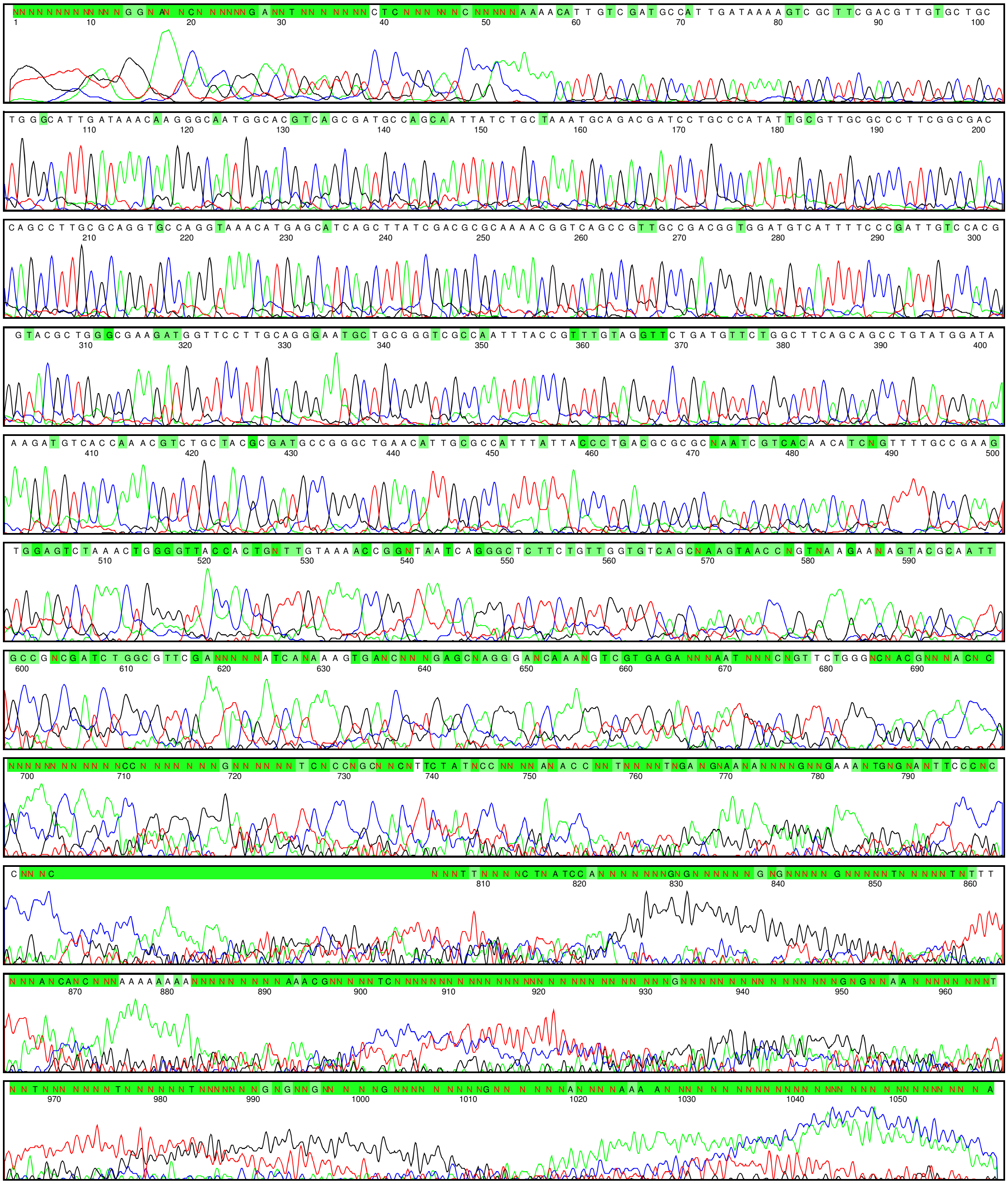

Unpurified4

Length: 1060
October 29, 2010 3:14:52 PM CDT

q>=20: 603
q>=30: 497
q>=40: 298

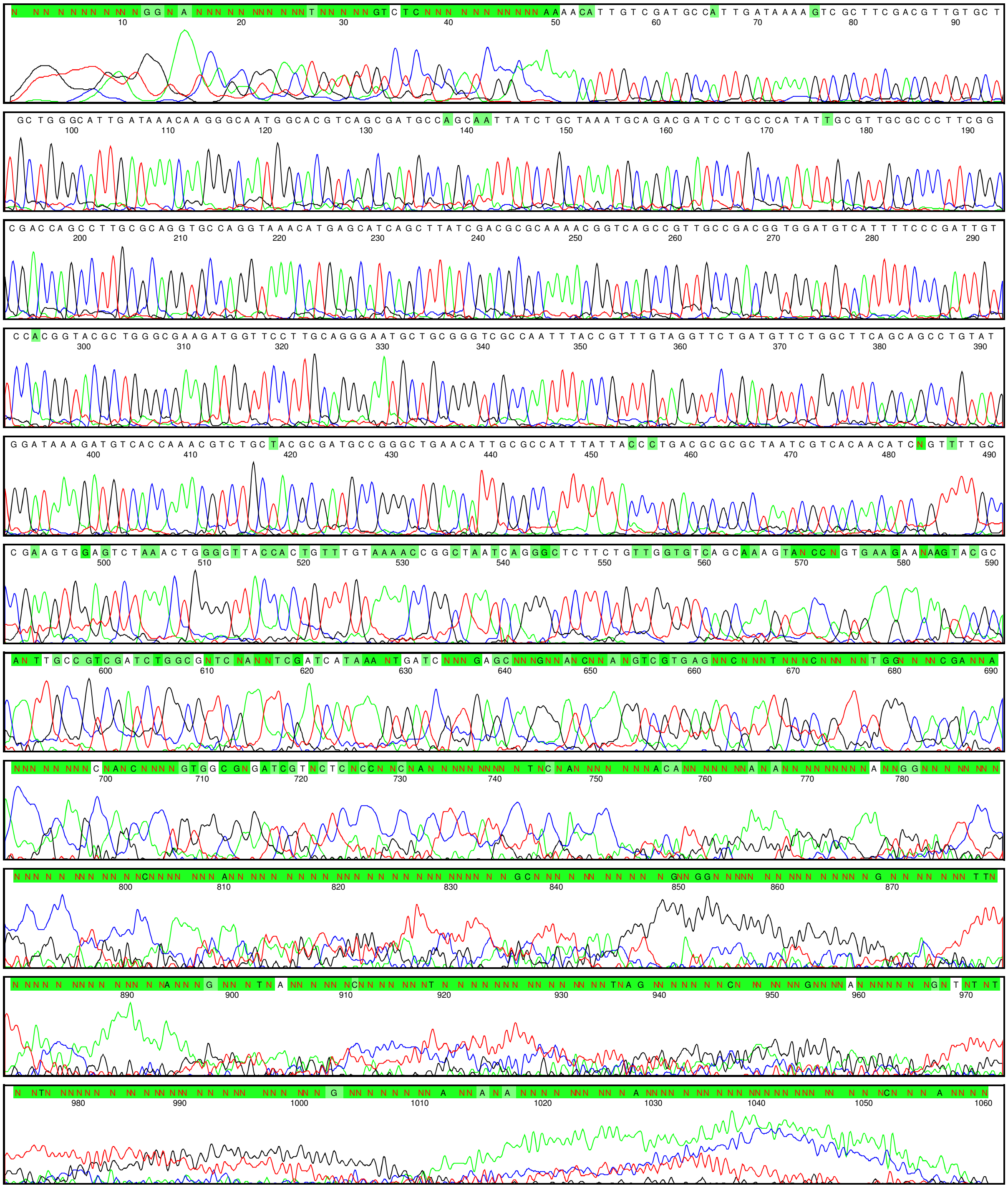

Supplement: Figure S3 — Raw sequence trace using NT primer. (5.62 MB PDF) [file pone.0014512.s003.pdf]
