# Supplementary material for: Removal of PCR Error Products and Unincorporated Primers by Metal-Chelate Affinity Chromatography
Source: PLoS One. 2011 Jan 14;6(1):e14512. doi: 10.1371/journal.pone.0014512 (PMC3021510; doi:10.1371/journal.pone.0014512)
Supplement: Figure S4 — ClustalW alignment of sequence data generated using NT and CT Primers. (0.08 MB DOC) [file pone.0014512.s004.doc]

ClustalW alignment of sequence data generated using NT Primer

where,

1-nt to 4-nt are unpurified samples

5-nt to 8-nt are IMAC purified samples and

9-nt to 12-nt are QIAquick purified samples.

CLUSTAL 2.0.12 multiple sequence alignment

6-nt -------NTNNNNNNNGGNNNNNGCGGNNNTGAAGTGTCTCTGCAATCGGCAAAAAACAT 53

8-nt ------NNNNNNNNNNGNAATCNGCGGANNTGAAGTGTCTCTGCAATCGGCAAAAAACAT 54

7-nt ---NNNNNNNNTNNNNGGNATNNGCGGANNTGAAGTGTCTCTGCAATCGGCAAAAAACAT 57

5-nt ---NNNNNNTNNNNNNGGNATCNGCGGANNTGAAGTGTCTCTGCAATCGGCAAAAAACAT 57

11-nt NNNNNNNNNNTNNGGNGGNANNNGCGGANNTGAAGTGTCTCTGCAATCGGCAAAAAACAT 60

12-nt -----NNNNNNNNNNNGNAANNNGCGGANNTGAAGTGTCTCTGCAATCGGCAAAAAACAT 55

9-nt ---NNNNNNNNNNNNGGNAANNNGCGGANNTGAAGTGTCTCTGCAATCGGCAAAAAACAT 57

10-nt -------NTNNNNNNNNGNANNNGCGGNNNTGAAGTGTCTCTGCAATCGGCAAAAAACAT 53

1-nt ---NNNNNNNNNNNNNGGNANCNNNNNGANNTNNNNNNNCTCNNNNNNNNNNNAAAACAT 57

2-nt ---NNNNNNNNNNNNNGGNANNNNNNNNANNTNNNNNNNCTCNNNNNNNNNNNAAAACAT 57

3-nt NNNNNNNNNNNNNGGNANNCNNNNNNGANNTNNNNNNNCTCNNNNNNCNNNNNAAAACAT 60

4-nt ----NNNNNNNNNNNGGNANNNNNNNNNNNTNNNNNGTCTCNNNNNNNNNNNAAAA-CAT 55

* * * * *** ***

6-nt TGTCGATGCCATTGATAAAAGTCGCTTCGACGTTGTGCTGCTGGGCATTGATAAACAAGG 113

8-nt TGTCGATGCCATTGATAAAAGTCGCTTCGACGTTGTGCTGCTGGGCATTGATAAACAAGG 114

7-nt TGTCGATGCCATTGATAAAAGTCGCTTCGACGTTGTGCTGCTGGGCATTGATAAACAAGG 117

5-nt TGTCGATGCCATTGATAAAAGTCGCTTCGACGTTGTGCTGCTGGGCATTGATAAACAAGG 117

11-nt TGTCGATGCCATTGATAAAAGTCGCTTCGACGTTGTGCTGCTGGGCATTGATAAACAAGG 120

12-nt TGTCGATGCCATTGATAAAAGTCGCTTCGACGTTGTGCTGCTGGGCATTGATAAACAAGG 115

9-nt TGTCGATGCCATTGATAAAAGTCGCTTCGACGTTGTGCTGCTGGGCATTGATAAACAAGG 117

10-nt TGTCGATGCCATTGATAAAAGTCGCTTCGACGTTGTGCTGCTGGGCATTGATAAACAAGG 113

1-nt TGTCGATGCCATTGATAAAAGTCGCTTCGACGTTGTGCTGCTGGGCATTGATAAACAAGG 117

2-nt TGTCGATGCCATTGATAAAAGTCGCTTCGACGTTGTGCTGCTGGGCATTGATAAACAAGG 117

3-nt TGTCGATGCCATTGATAAAAGTCGCTTCGACGTTGTGCTGCTGGGCATTGATAAACAAGG 120

4-nt TGTCGATGCCATTG-TAAAAGTCGCTTCGACGTTGTGCTGCTGGGCATTGATAAACAAGG 114

************** *********************************************

6-nt GCAATGGCACGTCAGCGATGCCAGCAATTATCTGCTAAATGCAGACGATCCTGCCCATAT 173

8-nt GCAATGGCACGTCAGCGATGCCAGCAATTATCTGCTAAATGCAGACGATCCTGCCCATAT 174

7-nt GCAATGGCACGTCAGCGATGCCAGCAATTATCTGCTAAATGCAGACGATCCTGCCCATAT 177

5-nt GCAATGGCACGTCAGCGATGCCAGCAATTATCTGCTAAATGCAGACGATCCTGCCCATAT 177

11-nt GCAATGGCACGTCAGCGATGCCAGCAATTATCTGCTAAATGCAGACGATCCTGCCCATAT 180

12-nt GCAATGGCACGTCAGCGATGCCAGCAATTATCTGCTAAATGCAGACGATCCTGCCCATAT 175

9-nt GCAATGGCACGTCAGCGATGCCAGCAATTATCTGCTAAATGCAGACGATCCTGCCCATAT 177

10-nt GCAATGGCACGTCAGCGATGCCAGCAATTATCTGCTAAATGCAGACGATCCTGCCCATAT 173

1-nt GCAATGGCACGTCAGCGATGCCAGCAATTATCTGCTAAATGCAGACGATCCTGCCCATAT 177

2-nt GCAATGGCACGTCAGCGATGCCAGCAATTATCTGCTAAATGCAGACGATCCTGCCCATAT 177

3-nt GCAATGGCACGTCAGCGATGCCAGCAATTATCTGCTAAATGCAGACGATCCTGCCCATAT 180

4-nt GCAATGGCACGTCAGCGATGCCAGCAATTATCTGCTAAATGCAGACGATCCTGCCCATAT 174

************************************************************

6-nt TGCGTTGCGCCCTTCGGCGACCAGCCTTGCGCAGGTGCCAGGTAAACATGAGCATCAGCT 233

8-nt TGCGTTGCGCCCTTCGGCGACCAGCCTTGCGCAGGTGCCAGGTAAACATGAGCATCAGCT 234

7-nt TGCGTTGCGCCCTTCGGCGACCAGCCTTGCGCAGGTGCCAGGTAAACATGAGCATCAGCT 237

5-nt TGCGTTGCGCCCTTCGGCGACCAGCCTTGCGCAGGTGCCAGGTAAACATGAGCATCAGCT 237

11-nt TGCGTTGCGCCCTTCGGCGACCAGCCTTGCGCAGGTGCCAGGTAAACATGAGCATCAGCT 240

12-nt TGCGTTGCGCCCTTCGGCGACCAGCCTTGCGCAGGTGCCAGGTAAACATGAGCATCAGCT 235

9-nt TGCGTTGCGCCCTTCGGCGACCAGCCTTGCGCAGGTGCCAGGTAAACATGAGCATCAGCT 237

10-nt TGCGTTGCGCCCTTCGGCGACCAGCCTTGCGCAGGTGCCAGGTAAACATGAGCATCAGCT 233

1-nt TGCGTTGCGCCCTTCGGCGACCAGCCTTGCGCAGGTGCCAGGTAAACATGAGCATCAGCT 237

2-nt TGCGTTGCGCCCTTCGGCGACCAGCCTTGCGCAGGTGCCAGGTAAACATGAGCATCAGCT 237

3-nt TGCGTTGCGCCCTTCGGCGACCAGCCTTGCGCAGGTGCCAGGTAAACATGAGCATCAGCT 240

4-nt TGCGTTGCGCCCTTCGGCGACCAGCCTTGCGCAGGTGCCAGGTAAACATGAGCATCAGCT 234

************************************************************

6-nt TATCGACGCGCAAAACGGTCAGCCGTTGCCGACGGTGGATGTCATTTTCCCGATTGTCCA 293

8-nt TATCGACGCGCAAAACGGTCAGCCGTTGCCGACGGTGGATGTCATTTTCCCGATTGTCCA 294

7-nt TATCGACGCGCAAAACGGTCAGCCGTTGCCGACGGTGGATGTCATTTTCCCGATTGTCCA 297

5-nt TATCGACGCGCAAAACGGTCAGCCGTTGCCGACGGTGGATGTCATTTTCCCGATTGTCCA 297

11-nt TATCGACGCGCAAAACGGTCAGCCGTTGCCGACGGTGGATGTCATTTTCCCGATTGTCCA 300

12-nt TATCGACGCGCAAAACGGTCAGCCGTTGCCGACGGTGGATGTCATTTTCCCGATTGTCCA 295

9-nt TATCGACGCGCAAAACGGTCAGCCGTTGCCGACGGTGGATGTCATTTTCCCGATTGTCCA 297

10-nt TATCGACGCGCAAAACGGTCAGCCGTTGCCGACGGTGGATGTCATTTTCCCGATTGTCCA 293

1-nt TATCGACGCGCAAAACGGTCAGCCGTTGCCGACGGTGGATGTCATTTTCCCGATTGTCCA 297

2-nt TATCGACGCGCAAAACGGTCAGCCGTTGCCGACGGTGGATGTCATTTTCCCGATTGTCCA 297

3-nt TATCGACGCGCAAAACGGTCAGCCGTTGCCGACGGTGGATGTCATTTTCCCGATTGTCCA 300

4-nt TATCGACGCGCAAAACGGTCAGCCGTTGCCGACGGTGGATGTCATTTTCCCGATTGTCCA 294

************************************************************

6-nt CGGTACGCTGGGCGAAGATGGTTCCTTGCAGGGAATGCTGCGGGTCGCCAATTTACCGTT 353

8-nt CGGTACGCTGGGCGAAGATGGTTCCTTGCAGGGAATGCTGCGGGTCGCCAATTTACCGTT 354

7-nt CGGTACGCTGGGCGAAGATGGTTCCTTGCAGGGAATGCTGCGGGTCGCCAATTTACCGTT 357

5-nt CGGTACGCTGGGCGAAGATGGTTCCTTGCAGGGAATGCTGCGGGTCGCCAATTTACCGTT 357

11-nt CGGTACGCTGGGCGAAGATGGTTCCTTGCAGGGAATGCTGCGGGTCGCCAATTTACCGTT 360

12-nt CGGTACGCTGGGCGAAGATGGTTCCTTGCAGGGAATGCTGCGGGTCGCCAATTTACCGTT 355

9-nt CGGTACGCTGGGCGAAGATGGTTCCTTGCAGGGAATGCTGCGGGTCGCCAATTTACCGTT 357

10-nt CGGTACGCTGGGCGAAGATGGTTCCTTGCAGGGAATGCTGCGGGTCGCCAATTTACCGTT 353

1-nt CGGTACGCTGGGCGAAGATGGTTCCTTGCAGGGAATGCTGCGGGTCGCCAATTTACCGTT 357

2-nt CGGTACGCTGGGCGAAGATGGTTCCTTGCAGGGAATGCTGCGGGTCGCCAATTTACCGTT 357

3-nt CGGTACGCTGGGCGAAGATGGTTCCTTGCAGGGAATGCTGCGGGTCGCCAATTTACCGTT 360

4-nt CGGTACGCTGGGCGAAGATGGTTCCTTGCAGGGAATGCTGCGGGTCGCCAATTTACCGTT 354

************************************************************

6-nt TGTAGGTTCTGATGTTCTGGCTTCAGCAGCCTGTATGGATAAAGATGTCACCAAACGTCT 413

8-nt TGTAGGTTCTGATGTTCTGGCTTCAGCAGCCTGTATGGATAAAGATGTCACCAAACGTCT 414

7-nt TGTAGGTTCTGATGTTCTGGCTTCAGCAGCCTGTATGGATAAAGATGTCACCAAACGTCT 417

5-nt TGTAGGTTCTGATGTTCTGGCTTCAGCAGCCTGTATGGATAAAGATGTCACCAAACGTCT 417

11-nt TGTAGGTTCTGATGTTCTGGCTTCAGCAGCCTGTATGGATAAAGATGTCACCAAACGTCT 420

12-nt TGTAGGTTCTGATGTTCTGGCTTCAGCAGCCTGTATGGATAAAGATGTCACCAAACGTCT 415

9-nt TGTAGGTTCTGATGTTCTGGCTTCAGCAGCCTGTATGGATAAAGATGTCACCAAACGTCT 417

10-nt TGTAGGTTCTGATGTTCTGGCTTCAGCAGCCTGTATGGATAAAGATGTCACCAAACGTCT 413

1-nt TGTAGGTTCTGATGTTCTGGCTTCAGCAGCCTGTATGGATAAAGATGTCACCAAACGTCT 417

2-nt TGTAGGTTCTGATGTTCTGGCTTCAGCAGCCTGTATGGATAAAGATGTCACCAAACGTCT 417

3-nt TGTAGGTTCTGATGTTCTGGCTTCAGCAGCCTGTATGGATAAAGATGTCACCAAACGTCT 420

4-nt TGTAGGTTCTGATGTTCTGGCTTCAGCAGCCTGTATGGATAAAGATGTCACCAAACGTCT 414

************************************************************

6-nt GCTACGCGATGCCGGGCTGAACATTGCGCCATTTATTACCCTGACGCGCGCTAATCGTCA 473

8-nt GCTACGCGATGCCGGGCTGAACATTGCGCCATTTATTACCCTGACGCGCGCTAATCGTCA 474

7-nt GCTACGCGATGCCGGGCTGAACATTGCGCCATTTATTACCCTGACGCGCGCTAATCGTCA 477

5-nt GCTACGCGATGCCGGGCTGAACATTGCGCCATTTATTACCCTGACGCGCGCTAATCGTCA 477

11-nt GCTACGCGATGCCGGGCTGAACATTGCGCCATTTATTACCCTGACGCGCGCTAATCGTCA 480

12-nt GCTACGCGATGCCGGGCTGAACATTGCGCCATTTATTACCCTGACGCGCGCTAATCGTCA 475

9-nt GCTACGCGATGCCGGGCTGAACATTGCGCCATTTATTACCCTGACGCGCGCTAATCGTCA 477

10-nt GCTACGCGATGCCGGGCTGAACATTGCGCCATTTATTACCCTGACGCGCGCTAATCGTCA 473

1-nt GCTACGCGATGCCGGGCTGAACATTGCGCCATTTATTACCCTGACGCGCGCTAATCGTCA 477

2-nt GCNNCGCGATGCCGGGCTGAACATTGCGCCATTTATTACCCTGACGCGCGCTAATCGTCA 477

3-nt GCTACGCGATGCCGGGCTGAACATTGCGCCATTTATTACCCTGACGCGCGCNAATCGTCA 480

4-nt GCTACGCGATGCCGGGCTGAACATTGCGCCATTTATTACCCTGACGCGCGCTAATCGTCA 474

** *********************************************** ********

6-nt CAACATCAGTTTTGCCGAAGTGGAGTCTAAACTGGGGTTACCACTGTTTGTAAAACCGGC 533

8-nt CAACATCAGTTTTGCCGAAGTGGAGTCTAAACTGGGGTTACCACTGTTTGTAAAACCGGC 534

7-nt CAACATCAGTTTTGCCGAAGTGGAGTCTAAACTGGGGTTACCACTGTTTGTAAAACCGGC 537

5-nt CAACATCAGTTTTGCCGAAGTGGAGTCTAAACTGGGGTTACCACTGTTTGTAAAACCGGC 537

11-nt CAACATCAGTTTTGCCGAAGTGGAGTCTAAACTGGGGTTACCACTGTTTGTAAAACCGGC 540

12-nt CAACATCAGTTTTGCCGAAGTGGAGTCTAAACTGGGGTTACCACTGTTTGTAAAACCGGC 535

9-nt CAACATCNGTTTTGCCGAAGTGGAGTCTAAACTGGGGTTACCACTGTTTGTAAAACCGGC 537

10-nt CAACATCAGTTTTGCCGAAGTGGAGTCTAAACTGGGGTTACCACTGTTTGTAAAACCGGC 533

1-nt CAACATCAGTTTTGCCGAAGTGGAGTCTAAACTGGGGTTACCACTGTTTGTAAAACCGGC 537

2-nt CAACATCAGTTTTGCCGAAGTGGAGNCTAAACTGGGGTTACCACTGTTTGTAAAACCGGC 537

3-nt CAACATCNGTTTTGCCGAAGTGGAGTCTAAACTGGGGTTACCACTGNTTGTAAAACCGGN 540

4-nt CAACATCNGTTTTGCCGAAGTGGAGTCTAAACTGGGGTTACCACTGTTTGTAAAACCGGC 534

******* ***************** ******************** ************

6-nt TAATCAGGGCTCTTCTGTTGGTGTCAGCAAAGTAACCAGTGAAGAACA-GTACGCAATTG 592

8-nt TAATCAGGGCTCTTCTGTTGGTGTCAGCAAAGTAACCAGTGAAGAACA-GTACGCAATTG 593

7-nt TAATCAGGGCTCTTCTGTTGGTGTCAGCAAAGTAACCAGTGAAGAACA-GTACGCAATTG 596

5-nt TAATCAGGGCTCTTCTGTTGGTGTCAGCAAAGTAACCAGTGAAGAACA-GTACGCAATTG 596

11-nt TAATCAGGGCTCTTCTGTTGGTGTCAGCAAAGTAACCAGTGAAGAACA-GTACGCAATTG 599

12-nt TAATCAGGGCTCTTCTGTTGGTGTCAGCAAAGTAACCAGTGAAGAACA-GTACGCAATTG 594

9-nt TAATCAGGGCTCTTCTGTTGGTGTCAGCAAAGTAACCAGTGAAGAACA-GTACGCAATTG 596

10-nt TAATCAGGGCTCTTCTGTTGGTGTCAGCAAAGTAACCAGTGAAGAACA-GTACGCAATTG 592

1-nt TAATCAGGGCTCTTCNNNNGGTGTCAGCAAAGTAACCAGTGAAGAACA-GTACGCANTTG 596

2-nt TAATCAGGGCTCTTCTGTTGGTGTCAGCAAAGTAACCAGTGAAGAACA-GTACGCAATTG 596

3-nt TAATCAGGGCTCTTCTGTTGGTGTCAGCNAAGTAACCNGTNAAGAANA-GTACGCAATTG 599

4-nt TAATCAGGGCTCTTCTGTTGGTGTCAGCAAAGTANCCNGTGAAGAANAAGTACGCANTTG 594

*************** ********* ***** ** ** ***** * ******* ***

6-nt CCGTCGATCTGGCGTTCGAATTCGATCATAAAGTGATCGTTGAGCAAGGGATC-AAAGGT 651

8-nt CCGTCGATCNNNCGTTCGAATTCGATCATAAAGTGATCGTTGAGCAAGGGATC-AAAGGT 652

7-nt CCGTCGATCTGGCGTTCGAATTCGATCATAAAGTGATCGTTGAGCAAGGGATC-AAAGGT 655

5-nt CCGTCGATCTGGCGTTCGAATTCGATCATAAAGTGATCGTTGAGCAAGGGATC-AAAGGT 655

11-nt CCGTCGATCTGGCGTTCGAATTCGATCATAAAGTGATCGTTGAGCAAGGGATC-AAAGGT 658

12-nt CCGTCGATCTGGCGTTCGAATTCGATCATAAAGTGATCGTTGAGCAAGGGATC-AAANGT 653

9-nt CCGTCGATCTGGCGTTCGAATTCGATCATAAAGTGATCGNTGAGCAAGGGATC-AAANGT 655

10-nt CCGTCGATCTGGCGTTCGAATTCGATCATAAAGTGATCNNTGAGCAAGGGATC-AAANGT 651

1-nt CCGTCGATCTGGCGTTCGAATTCGATCATAAAGTGATCGTTGAGCAAGGGATC-AAAGGT 655

2-nt CCGTCGATCTGGCGTTCGAATTCNATCATAAAGTGATCNNNGAGCAAGGNATC-NAAGGT 655

3-nt CCGNCGATCTGGCGTTCGANNNNNATCANAAAGTGANCNNNGAGCNAGGGANC-AAANGT 658

4-nt CCGTCGATCTGGCGNTCNANNTCGATCATAAANTGATCNNNGAGCNNNGNNANCNNANGT 654

*** ***** ** ** * **** *** *** * **** * * **

6-nt CGTGAGATCGAATGCGCAGTTCTGGGCAA-CGACAACCCGCAAGCCAGCACCTGTGGCGA 710

8-nt CGTGAGATCGAATGCGCAGTTCTGGGCAA-CGACNACCCGCAAGCCAGCACCTGTGGCGA 711

7-nt CGTGAGATCGAATGCGCAGTTCTGGGCAA-CGACAACCCGCAAGCCAGCACCTGTGGCGA 714

5-nt CGTGAGATCGAATGCGCAGTTCTGGGCAA-CGACAACCCGCAAGCCAGCACCTGTGGNGA 714

11-nt CGTGAGATCGAATGCGCAGTTCTGGGCAA-CGACNACCCGCAAGCCAGCACCTGTGGCGA 717

12-nt CGTGAGATCGAATGCGCAGTTCTGGGCAA-CGACNACCCGCAAGCCAGCACCTGTGGCGA 712

9-nt CGTGAGATCGAATGCGCANTTCTGGGCNN-CGACNACCCGCAAGCCANNNCCTGTGGCGA 714

10-nt CGTGAGATCGAATGCGCAGTTCTGGGCNA-CGACNACCCGCAAGCCANNNCCTGTGGCGA 710

1-nt CGTGAGATCNAATGCGCNGTTCTGGGCNA-CGACNACCCGCAAGCCANCACCTGTGGCGA 714

2-nt CGTGAGATCGAATGCGCNGTTCTGGGCNA-CGACNACCCGCNAGCCAGCACCTGTGGNGA 714

3-nt CGTGAGANNNAATNNNCNGTTCTGGGNCNACGNNNACNCNNNNNNNNNNNNCCNNNNNNN 718

4-nt CGTGAGNNCNNNTNNNCNNNNNTGGNNNN-CGANNANNNNNNNNCNANCNNNNGTGGCGN 713

****** * * *** ** *

6-nt GATCGTACTCACCAGCGACTTCTATGCCTACGACACCAAGTACATTGACGAAGANGGCGC 770

8-nt GATCGTACTCACCAGCGACTTCTATGCCTACGACACCAAGTACATTGACGAAGATGGCGC 771

7-nt GATCGTACTCACCAGCGACTTCTATGCCTACGACACCNAGTACATTGACGAAGATGGCGC 774

5-nt GATCGTACTCACCAGCGACTTCTATGCCTACGACACCAAGTACATTGACGAAGATGGCGC 774

11-nt GATCGTACTCACCAGCGACTTCTATGCCTACGANACCNAGTACATTGACGAANANGGCGC 777

12-nt GATCGTACTCACCNGCGACTTCTATGCCTACGACACCNAGTACATTGACGAANATGGNGC 772

9-nt GATCGTACTCANCNGCGACTTCTATGNCTACGANACCNAGTACATTGACGAANANGGCGC 774

10-nt GATCGTACTCACCAGCGACTTCTATGNCTACGANACCNAGTACATTGACGAANANGGNGC 770

1-nt GATCGTACTCACCAGCGANTTCTATGCCTACGACACCAAGTACNTTGANNAANANGGNGN 774

2-nt GATCGTANTCACCAGCGACTTNTATNCCTACGACANCAAGTNCNTTGANGAANANGNNNN 774

3-nt GNNNNNNTCNCCNGCNNCNTTCTATNCCNNNNANACCNNTNNNNTNGANGNAANANNNNG 778

4-nt GATCGTNCTCNCCNNCNANNNNNNNNNNTNCNANNNNNNNACANNNNNNNANANNNNNNN 773

* *

6-nt GAAAGTGGNAGNNNCC-GGCAGCCATTGC-GNCNGAAATCAACGATAAGATCCGGG-CGA 827

8-nt GAAAGTGGTAGTTCC--GGCAGCCATTGC-GCCAGAAATCAACGATAAGATCCGGG-CGA 827

7-nt GAAAGTGGTAGTTCC--GGCAGCCATTGC-GCCAGAAATCAACGATAAGATCCGGG-CGA 830

5-nt GAAAGTGNNAGTTCC--GGCAGCCATTGC-GCCAGAAATCAACGATAAGANCCGGG-CGN 830

11-nt GAAAGTGGTAGTTCC--NGCNGCCATTGC-GCCNGAAATCAACGATNAGATNCGGG-CGA 833

12-nt GAAAGTGGTAGTTCC--NGCAGCCATTGC-GCCNNAAATCNACGATNAGATCCNGG-CGA 828

9-nt GAAAGNGGNAGTTCC--NGCNGCCNTTGN-GCNNGAAATCANCGATNAGATCCNGGGCGA 831

10-nt GAAAGTGGNAGTTNC--NGCNGCCATTGC-GCCNGAAATCNNCGATNNNATCCNGGCGAN 827

1-nt NNAANNNNNNNNTNNN-NNCNNNNNNNNNCNNCNNANNNCNACNATAANATCNGGG-NNN 832

2-nt NAAANNNNNANNTTCCNNNNNNCCNNNNCNNCNNNANANCNACNATNAGATNNCGGNNNA 834

3-nt NNGAAANTGNGNANT--TCCCNCCNNNCNNNTTNNNNCTNATCCANNNNNNNGNGNNNNN 836

4-nt NNANNGGNNNNNNNN--NNNNNNNNNNNNCNNNNNNNANNNNNNNNNNNNNNNNNNNNNN 831

6-nt TTGCCGNTCAGGCTTATCAAACG-TTGGGATGCGCAGG-CATGGCGCGTGTAGACGTGTT 885

8-nt TTGNCGTTCAGGCTTATCAAACG-TTGGGATGCGCAGG-NATGGCGCGTGTAGACGTNNT 885

7-nt TTGNCGTTCNGGCTTATCAAACG-NTGGGANGCGCAGG-CATGGCGCGTGTAGACGTGTT 888

5-nt NTGNCGTTCAGGCTTNTCAAACG-TTGGGATGCNCAGG-NNTGGCGCGNGTAGACGTGTT 888

11-nt NTGCCGNTCNNNNNNNTCAAACG-TTGGGATGCNCNNGGNATGGCGCGNGNANACNTGTT 892

12-nt NTGCCGNTCNNGNTTATCANNNN-TTGGGATGCGCNNG-NNTGGCGCGNGNNNACNTGTT 886

9-nt NNGNNNNNCNNNNNTNNNNANANGTNNNGANGCNCNGG-NNNGGCGCGNGNNNNNCNTGT 890

10-nt TGNCNNNNNNNNNTTATCNAACGTTGGGGANGCGCANN-NNTGGCGCGNGNANNCNTGNT 886

1-nt NTGNNNTTCNNGCTTNTNANACG-TTGGGATGNGNNNGNNNNGGNNNNNGNNGNCGTGTT 891

2-nt TNGNNGTTNNNGNNNNNNNNAANNGNNGNNNNNNNNNNNNGNNNNNNNNNNGNCNTNNTT 894

3-nt NGNGNNNNNGNNNNNTNNNNNTNTTTNNNANCANCNNNAAAAAAA-ANNNNNNNNNAAAC 895

4-nt NNNGCNNNNNNNNNNNGNNGGNNNNNNNNNNNNNNNGNNNNNNNTTNNNNNNNNNNNNNN 891

6-nt TTT--AACCCCNGAAAACGAANNGGTGATCNACGA-GATCANNACACTNNCTGGCTNNNN 942

8-nt TTT--AACCCCAGAAAACGAAGTGGTGATCAACGA-GATCANCNCACTGNCTGGCTTCAN 942

7-nt TTT--AACCCCAGAAAACGAAGTGGTGATCAACGA-GATCANCACACTGNCTGGCTTCAC 945

5-nt TTT--AACCCCAGAAAACGAAGNGGTGATCAACGA-GATCANCACNNTGCCNGGCTTCAC 945

11-nt TTT--AACNCCAGANAACGAAGTGNTGANCAACNA-NATCNACANNCTGNCNNNNTTCNN 949

12-nt TTT--AACNNCNNNNAACGNANTGGTGANCAACNNANATCNACNCNCTGNCTGGCTTCNC 944

9-nt TTTTAANNNNCNNNNAACGANNNGGTNATCAACNA-NATCNNNNNNCTGNCTNNNNTCNC 949

10-nt TTT--AANNNCNGANAACGAANNGNNGNNCAACNA-NATCANNNNNCTGCNTGGNNTCNC 943

1-nt TTTTNNCCCCNNAAAAANNANNTGGTGATNAANNN-NATNNNNCNCNNNCNNGNNTTCNN 950

2-nt TTNANCCCCANAAAAANNNAANNGGNNNNAAACGA-GATNNNCNCNCTNNCNNNNNNTNN 953

3-nt GNN--NNNTCNNNNNNNNNNNNNNNNNNNNNNNNN--NNNNGNNNNNNNNNNNNNNNNGN 951

4-nt NAN--NNGNNNTNANNNNNNCNNNNNNNTNNNNNN--NNNNNNNNNTNAGNNNNNNNCNN 947

6-nt NNNCATCAGTNNGNNTCCNAANC-TGTGGCAANNCCAGCGGNCTGGGTTANNCCGATCTG 1001

8-nt CNNCATCAGTATGTATNCNAAGC-TGNNGNANGNC-AGCNGNCTGGGNNNNNCCGATCTG 1000

7-nt CAACATCAGTATGTATCCNNNN--TGTGGNAAGCC--AGNNGNNNGGNNNNNNCGANCTG 1001

5-nt CAANNNCANNNTGNNTCCNAAGC-TGNNGNAAGNC-AGCGGNCTGGGNNNNCCNNNN-TG 1002

11-nt CNACNNCANNNTGTATNCNNNNC-TGTGGNAAGNC-AGCGNNCNGGNNNNNNNCGATNNG 1007

12-nt CANNNTCANNNTGTATCCNNANC-TGNGGCANNNC--NGCGGNNNGNNTNCNNCNANNNG 1001

9-nt CNNCNNCNNNNTNNATNCNAANC-TGTGGCAANNN--NCNNNNNGGGNTNCANCNANNNG 1006

10-nt CANCNTCANNNTGTATNCNNANNCTGNGGCAANNN--GCNGNNNGGGNTNNNNCNATNNN 1001

1-nt NNNNNNNNNNTNNNNCCNANNCN--NNNGNNAGCC-AGNNGNNNGNNNNANNNNNNNNNN 1007

2-nt NNNTNNTNNNNNNNGNNANNNNNANNNNGNNNNNNNNNNGNNNGNNNNGNNNNNNNNNNT 1013

3-nt GNNAANNNNNNNNTNNTNNNNNN--NTNNNNNNTN---NNNNNNGNGNNGNNNNNGNNNN 1006

4-nt NNNNNGNNNNANNNNNNNGNTNT--NTNNTNNNNN---NNNNNNNNNNNNNNNNNNNNNG 1002

6-nt NNCNNNCNTTT-GATTNNNCTG-GNNCTGGANCGTCNCGNNNCNNNNNNNNNNNNNNANC 1059

8-nt ATCNCGCNTTN-GANTNNNCTG-GCGCTGGANCGTCNCNNTNNNGNTNANNNANNNNANN 1058

7-nt ATCNCNCGNTN-GATNNGCT---GNNCTGGNNNGTCNNNCTNNNNNNANNNANNNANANN 1057

5-nt ATNNCNCGTTT-GATTGANCTG-NNNNNGGANCNNNNNNNNNCNNNNNNNNNNANNNNAA 1060

11-nt ATCNCGCNNTTTGATTGANCNN-NNGCTGNNNNNNNNCGNNNNNNNNNNNNNANNNAAAA 1066

12-nt ATCNNNCNNTTTGNNTGNNCNNGNNGCTGNNNCGNNNNGNNNNNGNNNANNNNNNNAAAA 1061

9-nt ATCNNNCNNTNNGATTNNNNNGNNCNNGNNNNNNNNNNNNNNNANNANTNNNNNNNAAAC 1066

10-nt ANCNNNCNNTTNNNTNNNNNGNNCTGNANNNNNNNNNNNNNCNGNNNANNNNNNNNNNAN 1061

1-nt NNNNNNNTNNTNNNNNGNNNNNNGNNNNNNNNNNNNNNNNNNNNNNNNNNNNNNNNNNNN 1067

2-nt NNNTNNNNNNNNNNNTNNNNNGNGNNNGNNNNNNNNNNNNNNNANTNNNAAANAANNNNN 1073

3-nt NNNNNGNNNNNNANNNNAAAANNNNNNNNNNNNNNNNNNNNNNNNNNNNNNNA------- 1059

4-nt NNNNNNNNANNANANNNNNNNNNNANNNNNNNNNNNNNNNNNNNNNNNCNNNANNNN--- 1059

* *

6-nt CNNNANGNANNNTTTTNNCNNCCNNNNNNCCCCNNNN--------------------- 1096

8-nt NNNANGNANNNTTTTNNNNCNNNNNNNNCCCCCNNA---------------------- 1094

7-nt NNNATNNNNNATTTNNNGCCNNGNANCCNNNANNNNN--------------------- 1094

5-nt ANNNNNNNNNNNNNNTTTNANNNCNNGNNNCCCCNNA--------------------- 1097

11-nt NCNNNANNNNANNANTNNNNNNNNNNNNNCCCCCNNNANN------------------ 1106

12-nt NNNCAATGNNANNNNNNNNNNNNNNNNNNCCCNNNNANA------------------- 1100

9-nt NNNNANGNNNNANTTNNNNNNNGNNNNNNCNCNNNNN--------------------- 1103

10-nt NNNNNNGNNNNNNNTNNNNNNNNNGNNNCCCCCNNNN--------------------- 1098

1-nt NNNNNNNNNNANCNCCNNNNNNN----------------------------------- 1090

2-nt NNNAANNNNNNNNNNNNNNCNNNNNNNNTTNNNTTTTNNTTTTTNNNNNNNNNNNNNN 1131

3-nt ----------------------------------------------------------

4-nt ----------------------------------------------------------

ClustalW alignment of sequence data generated using CT Primer

where,

1-ct to 4-ct are unpurified samples

5-nt to 8-nt are IMAC purified samples and

9-nt to 12-nt are QIAquick purified samples.

CLUSTAL 2.0.12 multiple sequence alignment

6-ct -------ANNNNNNNNNATCCNCAGCGTGNCGCTCCNGCGCCNGCTCAATCAAA-CGCGT 52

8-ct ------NNNNNNNNNNNNNCCNNAGCGTGNCGCTCCNGCGCCNGCTCAATCAAA-CGCGT 53

5-ct -------ANNNNNNNNTATCCGCAGCGTGNCGCTCCNGCGCCNGCTCAATCAAA-CGCGT 52

7-ct ---------NNNNNNNNNNCCGNAGCGTGNCGCTCCNGCGCCNGCTCAATCAAA-CGCGT 50

11-ct ---------NNNNNNNNNNCNNNAGCGTGACGCTCNNGCGCCAGCTCAATCAAA-CGCGT 50

10-ct --------NNNNNNNNNANCCGCAGCGTGACGCTCNNGCGCCNGCTCAATCAAA-CGCGT 51

12-ct -------NNNNNNNNNNANCCGCAGCGTGNNGCTCNNGCGCCNGCTCAATCAAA-CGCGT 52

9-ct -------NNNNNNNNNNANCCGCAGCGTGNCGCTCNNGCGCCAGCTCAATCAAA-CGCGT 52

3-ct -----NNNNNNNNNNNNNCNNNNNNNNNNNNGCNNNNNNNCNANNNNNANNNNAACGCGT 55

1-ct NNNNNNNNNNNNNNNNNNNNNNNNNNNNNNNNCTNNNNNNCCNNNNNNNNNNNAACGCGT 60

2-ct --NNNNNNNNNNNNGNNNNCNNNNNNNNNNNNCNNNNNNNNCNNNNNNNNNNNAACGCGT 58

4-ct ---NNNNNNNNNNNNNCNNNNNNNNNNNNNNCNNNNNNNCCNNNNNNNNNNNNAACGCGT 57

***** * * * *****

6-ct GATCAGATCGGTGTAACCCAGACCGCTGGCTTGCCACAGCTTCGGATACATACTGATGTT 112

8-ct GATCAGATCGGTGTAACCCAGACCGCTGGCTTGCCACAGCTTCGGATACATACTGATGTT 113

5-ct GATCAGATCGGTGTAACCCAGACCGCTGGCTTGCCACAGCTTCGGATACATACTGATGTT 112

7-ct GATCAGATCGGTGTAACCCAGACCGCTGGCTTGCCACAGCTTCGGATACATACTGATGTT 110

11-ct GATCAGATCGGTGTAACCCAGACCGCTGGCTTGCCACAGCTTCGGATACATACTGATGTT 110

10-ct GATCAGATCGGTGTAACCCAGACCGCTGGCTTGCCACAGCTTCGGATACATACTGATGTT 111

12-ct GATCAGATCGGTGTAACCCAGACCGCTGGCTTGCCACAGCTTCGGATACATACTGATGTT 112

9-ct GATCAGATCGGTGTAACCCAGACCGCTGGCTTGCCACAGCTTCGGATACATACTGATGTT 112

3-ct GATCAGATCGGTGTAACCCAGACCGCTGGCTTGCCACAGCTTCGGATACATACTGATGTT 115

1-ct GATCAGATCGGTGTAACCCAGACCGCTGGCTTGCCACAGCTTCGGATACATACTGATGTT 120

2-ct GATCAGATCGGTGTAACCCAGACCGCTGGCTTGCCACAGCTTCGGATACATACTGATGTT 118

4-ct GATCAGATCGGTGTAACCCAGACCGCTGGCTTGCCACAGCTTCGGATACATACTGATGTT 117

************************************************************

6-ct GGTGAAGCCAGGCAGTGTGTTGATCTCGTTGATCACCACTTCGTTTTCTGGGGTTAAAAA 172

8-ct GGTGAAGCCAGGCAGTGTGTTGATCTCGTTGATCACCACTTCGTTTTCTGGGGTTAAAAA 173

5-ct GGTGAAGCCAGGCAGTGTGTTGATCTCGTTGATCACCACTTCGTTTTCTGGGGTTAAAAA 172

7-ct GGTGAAGCCAGGCAGTGTGTTGATCTCGTTGATCACCACTTCGTTTTCTGGGGTTAAAAA 170

11-ct GGTGAAGCCAGGCAGTGTGTTGATCTCGTTGATCACCACTTCGTTTTCTGGGGTTAAAAA 170

10-ct GGTGAAGCCAGGCAGTGTGTTGATCTCGTTGATCACCACTTCGTTTTCTGGGGTTAAAAA 171

12-ct GGTGAAGCCAGGCAGTGTGTTGATCTCGTTGATCACCACTTCGTTTTCTGGGGTTAAAAA 172

9-ct GGTGAAGCCAGGCAGTGTGTTGATCTCGTTGATCACCACTTCGTTTTCTGGGGTTAAAAA 172

3-ct GGTGAAGCCAGGCAGTGTGTTGATCTCGTTGATCACCACTTCGTTTTCTGGGGTTAAAAA 175

1-ct GGTGAAGCCAGGCAGTGTGTTGATCTCGTTGATCACCACTTCGTTTTCTGGGGTTAAAAA 180

2-ct GGTGAAGCCAGGCAGTGTGTTGATCTCGTTGATCACCACTTCGTTTTCTGGGGTTAAAAA 178

4-ct GGTGAAGCCAGGCAGTGTGTTGATCTCGTTGATCACCACTTCGTTTTCTGGGGTTAAAAA 177

************************************************************

6-ct CACGTCTACACGCGCCATGCCTGCGCATCCCAACGTTTGATAAGCCTGAACGGCAATCGC 232

8-ct CACGTCTACACGCGCCATGCCTGCGCATCCCAACGTTTGATAAGCCTGAACGGCAATCGC 233

5-ct CACGTCTACACGCGCCATGCCTGCGCATCCCAACGTTTGATAAGCCTGAACGGCAATCGC 232

7-ct CACGTCTACACGCGCCATGCCTGCGCATCCCAACGTTTGATAAGCCTGAACGGCAATCGC 230

11-ct CACGTCTACACGCGCCATGCCTGCGCATCCCAACGTTTGATAAGCCTGAACGGCAATCGC 230

10-ct CACGTCTACACGCGCCATGCCTGCGCATCCCAACGTTTGATAAGCCTGAACGGCAATCGC 231

12-ct CACGTCTACACGCGCCATGCCTGCGCATCCCAACGTTTGATAAGCCTGAACGGCAATCGC 232

9-ct CACGTCTACACGCGCCATGCCTGCGCATCCCAACGTTTGATAAGCCTGAACGGCAATCGC 232

3-ct CACGTCTACACGCGCCATGCCTGCGCATCCCAACGTTTGATAAGCCTGAACGGCAATCGC 235

1-ct CACGTCTACACGCGCCATGCCTGCGCATCCCAACGTTTGATAAGCCTGAACGGCAATCGC 240

2-ct CACGTCTACACGCGCCATGCCTGCGCATCCCAACGTTTGATAAGCCTGAACGGCAATCGC 238

4-ct CACGTCTACACGCGCCATGCCTGCGCATCCCAACGTTTGATAAGCCTGAACGGCAATCGC 237

************************************************************

6-ct CCGGATCTTATCGTTGATTTCTGGCGCAATGGCTGCCGGAACTACCACTTTCGCGCCATC 292

8-ct CCGGATCTTATCGTTGATTTCTGGCGCAATGGCTGCCGGAACTACCACTTTCGCGCCATC 293

5-ct CCGGATCTTATCGTTGATTTCTGGCGCAATGGCTGCCGGAACTACCACTTTCGCGCCATC 292

7-ct CCGGATCTTATCGTTGATTTCTGGCGCAATGGCTGCCGGAACTACCACTTTCGCGCCATC 290

11-ct CCGGATCTTATCGTTGATTTCTGGCGCAATGGCTGCCGGAACTACCACTTTCGCGCCATC 290

10-ct CCGGATCTTATCGTTGATTTCTGGCGCAATGGCTGCCGGAACTACCACTTTCGCGCCATC 291

12-ct CCGGATCTTATCGTTGATTTCTGGCGCAATGGCTGCCGGAACTACCACTTTCGCGCCATC 292

9-ct CCGGATCTTATCGTTGATTTCTGGCGCAATGGCTGCCGGAACTACCACTTTCGCGCCATC 292

3-ct CCGGATCTTATCGTTGATTTCTGGCGCAATGGCTGCCGGAACTACCACTTTCGCGCCATC 295

1-ct CCGGATCTTATCGTTGATTTCTGGCGCAATGGCTGCCGGAACTACCACTTTCGCGCCATC 300

2-ct CCGGATCTTATCGTTGATTTCTGGCGCAATGGCTGCCGGAACTACCACTTTCGCGCCATC 298

4-ct CCGGATCTTATCGTTGATTTCTGGCGCAATGGCTGCCGGAACTACCACTTTCGCGCCATC 297

************************************************************

6-ct TTCGTCAATGTACTTGGTGTCGTAGGCATAGAAGTCGCTGGTGAGTACGATCTCGCCACA 352

8-ct TTCGTCAATGTACTTGGTGTCGTAGGCATAGAAGTCGCTGGTGAGTACGATCTCGCCACA 353

5-ct TTCGTCAATGTACTTGGTGTCGTAGGCATAGAAGTCGCTGGTGAGTACGATCTCGCCACA 352

7-ct TTCGTCAATGTACTTGGTGTCGTAGGCATAGAAGTCGCTGGTGAGTACGATCTCGCCACA 350

11-ct TTCGTCAATGTACTTGGTGTCGTAGGCATAGAAGTCGCTGGTGAGTACGATCTCGCCACA 350

10-ct TTCGTCAATGTACTTGGTGTCGTAGGCATAGAAGTCGCTGGTGAGTACGATCTCGCCACA 351

12-ct TTCGTCAATGTACTTGGTGTCGTAGGCATAGAAGTCGCTGGTGAGTACGATCTCGCCACA 352

9-ct TTCGTCAATGTACTTGGTGTCGTAGGCATAGAAGTCGCTGGTGAGTACGATCTCGCCACA 352

3-ct TTCGTCAATGTACTTGGTGTCGTAGGCATAGAAGTCGCTGGTGAGTACGATCTCGCCACA 355

1-ct TTCGTCAATGTACTTGGTGTCGTAGGCATAGAAGTCGCTGGTGAGTACGATCTCGCCACA 360

2-ct TTCGTCAATGTACTTGGTGTCGTAGGCATAGAAGTCGCTGGTGAGTACGATCTCGCCACA 358

4-ct TTCGTCAATGTACTTGGTGTCGTAGGCATAGAAGTCGCTGGTGAGTACGATCTCGCCACA 357

************************************************************

6-ct GGTGCTGGCTTGCGGGTTGTCGTTGCCCAGAACTGCGCATTCGATCTCACGACCTTTGAT 412

8-ct GGTGCTGGCTTGCGGGTTGTCGTTGCCCAGAACTGCGCATTCGATCTCACGACCTTTGAT 413

5-ct GGTGCTGGCTTGCGGGTTGTCGTTGCCCAGAACTGCGCATTCGATCTCACGACCTTTGAT 412

7-ct GGTGCTGGCTTGCGGGTTGTCGTTGCCCAGAACTGCGCATTCGATCTCACGACCTTTGAT 410

11-ct GGTGCTGGCTTGCGGGTTGTCGTTGCCCAGAACTGCGCATTCGATCTCACGACCTTTGAT 410

10-ct GGTGCTGGCTTGCGGGTTGTCGTTGCCCAGAACTGCGCATTCGATCTCACGACCTTTGAT 411

12-ct GGTGCTGGCTTGCGGGTTGTCGTTGCCCAGAACTGCGCATTCGATCTCACGACCTTTGAT 412

9-ct GGTGCTGGCTTGCGGGTTGTCGTTGCCCAGAACTGCGCATTCGATCTCACGACCTTTGAT 412

3-ct GGTGCTGGCTTGCGGGTTGTCGTTGCCCAGAACTGCGCATTCGATCTCACGACCTTTGAT 415

1-ct GGTGCTGGCTTGCGGGTTGTCGTTGCCCAGAACTGCGCATTCGATCTCACGACCTTTGAT 420

2-ct GGTGCTGGCTTGCGGGTTGTCGTTGCCCAGAACTGCGCATTCGATCTCACGACCTTTGAT 418

4-ct GGTGCTGGCTTGCGGGTTGTCGTTGCCCAGAACTGCGCATTCGATCTCACGACCTTTGAT 417

************************************************************

6-ct CCCTTGCTCAACGATCACTTTATGATCGAATTCGAACGCCAGATCGACGGCAATTGCGTA 472

8-ct CCCTTGCTCAACGATCACTTTATGATCGAATTCGAACGCCAGATCGACGGCAATTGCGTA 473

5-ct CCCTTGCTCAACGATCACTTTATGATCGAATTCGAACGCCAGATCGACGGCAATTGCGTA 472

7-ct CCCTTGCTCAACGATCACTTTATGATCGAATTCGAACGCCAGATCGACGGCAATTGCGTA 470

11-ct CCCTTGCTCAACGATCACTTTATGATCGAATTCGAACGCCAGATCGACGGCAATTGCGTA 470

10-ct CCCTTGCTCAACGATCACTTTATGATCGAATTCGAACGCCAGATCGACGGCAATTGCGTA 471

12-ct CCCTTGCTCAACGATCACTTTATGATCGAATTCGAACGCCAGATCGACGGCAATTGCGTA 472

9-ct CCCTTGCTCAACGATCACTTTATGATCGAATTCGAACGCCAGATCGACGGCAATTGCGTA 472

3-ct CCCTTGCTCAACGATCACTTTATGATCGAATTCGAACGCCAGATCGACGGCAATTGCGTA 475

1-ct CCCTTGCTCAACGATCACTTTATGATCGAATTCGAACGCCAGATCGACGGCNATTGCGTA 480

2-ct CCCTTGCTCAACGATCACTTTATGATCGAATTCGAACGCCAGATCGACGGCAATTGCGTA 478

4-ct CCCTTGCTCAACGATCACTTTATGATCGAATTCGAACGCCAGATCGACGGCAATTGCGTA 477

*************************************************** ********

6-ct CTGTTCTTCACTGGTTACTTTGCTGACACCAACAGAAGAGCCCTGATTAGCCGGTTTTAC 532

8-ct CTGTTCTTCACTGGTTACTTTGCTGACACCAACAGAAGAGCCCTGATTAGCCGGTTTTAC 533

5-ct CTGTTCTTCACTGGTTACTTTGCTGACACCAACAGAAGAGCCCTGATTAGCCGGTTTTAC 532

7-ct CTGTTCTTCACTGGTTACTTTGCTGACACCAACAGAAGAGCCCTGATTAGCCGGTTTTAC 530

11-ct CTGTTCTTCACTGGTTACTTTGCTGACACCAACAGAAGAGCCCTGATTAGCCGGTTTTAC 530

10-ct CTGTTCTTCACTGGTTACTTTGCTGACACCAACAGAAGAGCCCTGATTAGCCGGTTTTAC 531

12-ct CTGTTCTTCACTGGTTACTTTGCTGACACCAACAGAAGAGCCCTGATTAGCCGGTTTTAC 532

9-ct CTGTTCTTCACTGGTTACTTTGCTGACACCAACAGAAGAGCCCTGATTAGCCGGTTTTAC 532

3-ct CTGTTCTTCACTGGTTACTTTGCTGACACCAACAGAAGAGCCCTGATTAGCCGGTTTTAC 535

1-ct CTGTTCTTCACTGGTTACTTTGCTGACACCNACAGAAGANCCCTGATTAGCCGGTTTTAC 540

2-ct CTGTTCTTCACTGGTTACTTTGCTGACACCNACAGAAGAGCCCTGATTAGCCGGNTTTAC 538

4-ct CTGTTCTTCACTGGTTACTTTGCTGACACCAACAGAAGAGCCCTGATTAGCCGGTTTTAC 537

****************************** ******** ************** *****

6-ct AAACAGTGGT-AACCCCNGTTTAGACTCCACTTCGGCAAAACTGATGTTGTGACGATTAG 591

8-ct AAACAGNGGT-AACCCCAGTTTAGACTCCACTTCGGCAAAACTGATGTTGTGACGATTAG 592

5-ct AAACAGTGGT-AACCCCAGTTTAGACTCCACTTCGGCAAAACTGATGTTGTGACGATTAG 591

7-ct AAACAGTGGT-AACCCCAGTTTAGACTCCACTTCGGCAAAACTGATGTTGTGACGATTAG 589

11-ct AAACAGTGGT-AACCCCAGTTTAGACTCCACTTCGGCAAAACTGATGTTGTGACGATTAG 589

10-ct AAACAGTGGT-AACCCCAGTTTAGACTCCACTTCGGCAAAACTGATGTTGTGACGATTAG 590

12-ct AAACAGTGGT-AACCCCAGTTTAGACTCCACTTCGGCAAAACTGATGTTGTGACGATTAG 591

9-ct AAACAGTGGT-AACCCCAGTTTAGACTCCACTTCGGCAAAACTGATGTTGTGACGATTAG 591

3-ct AAACAGTGGT-AACCCCAGTTTAGACTCCACTTCGGCAAAACTGATGTTGTGACGATTAG 594

1-ct AAANNGTGNT-AACCCCNGTTTAGACTCCACTTCGGCNAAACTGATGTTGTGACGATTAG 599

2-ct AAACNGNGNNTANNCCCNGTTTAGACTCNACTTCGGCAAAACTGANGTTGTGACGATNAG 598

4-ct AAACAGTGGN-NACCCCAGTTTAGACTCCACTTCGGCAAAACTGANGTTGTGACGATTAG 596

*** * * *** ********** ******** ******* *********** **

6-ct CGCGCGTCAGGGTAATAAATGGCGCAATGTTCAGCCCGGC-ATCGCGTAGCAGACGTTTG 650

8-ct CGCGCGTCAGGGTAATAAATGGCGCAATGTTCAGCCCGGC-ATCGCGTAGCAGACGTTTG 651

5-ct CGCGCGTCAGGGTAATAAATGGCGCAATGTTCAGCCCGGC-ATCGCGTAGCAGACGTTTG 650

7-ct CGCGCGTCAGGGTAATAAATGGCGCAATGTTCAGCCCGGC-ATCGCGTAGCAGACGTTTG 648

11-ct CGCGCGTCAGGGTAATAAATGGCGCAATGTTCAGCCCGGC-ATCGCGTAGCAGACGTTTG 648

10-ct CGCGCGTCAGGGTAATAAATGGCGCAATGTTCAGCCCGGC-ATCGCGTAGCAGACGTTTG 649

12-ct CGCGCGTCAGGGTAATAAATGGCGCAATGTTCAGCCCGGC-ATCGCGTAGCAGACGTTTG 650

9-ct CGCGCGTCAGGGTAATAAATGGCGCAATGTTCAGCCCGGC-ATCGCGTAGCANACGTTTG 650

3-ct CGCGCGTCAGGGTAATAAATGGCGCAATGTTCAGCCCGGC-ATCGCGTAGCAGACGTTTG 653

1-ct CGCGCGTCAGGGTAATANNNNGCGCAATGTTCAGCCCNGNCATCGCGTAGCANANGTTTN 659

2-ct NNCGCGTCNNNGTAATAAATGNNGCAATGTTCAGCCCGNC-ATCGCGNANCAGANGTTTG 657

4-ct CGCGCGTCAGGNTAATAAATGGCGCAATGTTCAGCCCGGC-ATCGCGTAGCAGACGTTTG 655

****** ***** ************** ****** * ** * ****

6-ct GTGACATCTTTATCC-ATACAGGC-TGCTGAAGCCAGAACATCAGAACCTACAAACGGTA 708

8-ct GTGACATCTTTATCC-ATACAGGC-TGCTGAAGCCAGANCATCANAACCTACNAACGGTA 709

5-ct GTGACATCTTTATCC-ATACAGGC-TGCTGAAGCCAGAACATCAGAACCTACAAACGGTA 708

7-ct GTGACATCTTTATCC-ATACAGGC-TGCTGAAGCCAGAACATCAGAACCTACAAACGGTA 706

11-ct GTGACATCTTTATCC-ATACAGGC-TGCTGAAGCCAGAACATCAGAACCTACAAACGGTA 706

10-ct GTGACATCTTTATCC-ATACAGGC-TGCTGAAGCCAGAACATCAGAACCTACAAACGGTA 707

12-ct GTGACATCTTTATCC-ATACAGGC-TGCTGAAGCCAGAACATCAGAACCTACAAACGGTA 708

9-ct GTGACATCTTTATCC-ATACAGGC-TGCTGAAGCCAGAACATCAGAACCTACNAACGGTA 708

3-ct GTGACATCTTTATNC-ATACAGGC-TGCTGAAGCCAGAACATCAGAACCTACAAACGGTA 711

1-ct NNGACNTCTTTNNNCCNTACNGGC-TGCTNNNNNCANAACATCAGAACNTACAAACGNNA 718

2-ct GTGACNTCTTTATCC-ATACNNGN-NGCTGAANCCNGAACATCNNAACCTACAAACGGNN 715

4-ct GTGACATCTTTATCC-ATACAGGNCTGCTGAAGCCAGAACATCAGAACCTACAAACGGNA 714

*** ***** * *** * *** * * **** *** *** ****

6-ct AATTGGCGACCCGCAGCATTCCCT-GCAAGGAACCATCTTCGCCCAGCGTACCGTGGACA 767

8-ct AATTGGCGACCCGCAGCATTCCCT-GCAAGGAACCATCTTCGCCCAGCGTACCGTGGACA 768

5-ct AATTGGCGACCCGCAGCATTCCCT-GCAAGGAACCATCTTCGCCCAGCGTACCGTGGACA 767

7-ct AATTGGCGACCCGCAGCATTCCCT-GCAAGGAACCATCTTCGCCCAGCGTACCGTGGACA 765

11-ct AATTGGCGACCCGCAGCATTCCCT-GCAAGGAACCATCTTCGCCCAGCGTACCGTGNACA 765

10-ct AATTGGCGACCCGCAGCATTCCCT-GCAAGGAACCATCTTCGCCCAGCGTACCGTGNACA 766

12-ct AATTGGCGACCCGCAGCATTCCCT-GCAAGGAACCATCTTCGCCCAGCGTACCGTGNACA 767

9-ct NATTGGCGACCCGCAGCATTCCCT-GCAAGGAACCNTCTTCGCCCAGCGTANNNTGNACA 767

3-ct AATTGGCGACCCGCAGCATTCCCT-GCAAGGNANCATCTTCGCCCAGCGTACNGTGGANA 770

1-ct AANTGGCGACCCGCAGCATTCCCTGNNAAGGAANCATCTTCGCCCAGCGNANCGNNGANN 778

2-ct AATTGNCGANCCGCANCATTNNCCTGCANGGANNCATCTTCGCCCAGNGNANNGNGGANN 775

4-ct AATTNGCGACCCGCAGCNNTNCCN-GCANNNNANNNTCTTCGCCCAGCGTANCGNGNNNA 773

* * *** ***** * * * * *********** * *

6-ct ATCGGGAAAATGACAT--CCACCGTCGGCAACGGCTG-ACCGTTTTGCGCGNCGATAAGC 824

8-ct ATCGGGAAAATGACAT--CCACCGTCGGCAACGGCTG-ACCGTTTTGCGCGTCGATAAGC 825

5-ct ATCGGGAAAATGACAT--CCACCGTCGGCAACGGCTG-ACCGTTTTGCGCGTCGATAAGC 824

7-ct ATCGGGAAAATGACAT--CCACCGTCGGCAACGGCTG-ACCGTTTTGCGCGTCGATAAGC 822

11-ct ATCGGGAAAATGACAT--CCACCGTCGGCAACGGCTG-ACCGTTTTGCGCGTCGANNAGC 822

10-ct ATCGGGAAAATGACAT--CCACCGTCGGNANCGGCTG-ANCGTTTTGCGCGTCGATAAGC 823

12-ct ATCGGGAAAATGACAT--CCACCGTCGGCAACGGCTG-ANCGTTTTGCGCGTCGANAAGC 824

9-ct ATCGGGAAAATGACAT--CCACCGTCGGCAACNGCTG-ANCGTTTTGNGCGTCGANNANC 824

3-ct ATCGGGAAAATGACAT--NCNCCGTCNGCANNGNCTG-ACCGTTTTGCGCGTCGATNAGC 827

1-ct ATNNGGGAAAATGNCNTCCNNCNNNCNNNNACNGNCNGANCGTTTNNNNCNTNNGNTNAN 838

2-ct NNNNNNAAAANNNNCNNCCCNCCNNCNNCNACGGCNG-ACCGTTTTGNNNNNCGNNNANN 834

4-ct ATCGGGAAAATGACNT--CCNCCNNCGGCANCNGCTN-ANCGTTTTGNGCGTCGATNANC 830

*** * * * *****

6-ct TGAT---GCTCATGTTTACCTGGCA-CCTGCGCAANGNTGGTC-GNCGNNNGG-CGCAAC 878

8-ct TGAT---GCTCATGNTTACCTGGCA-CCTGCGCAAGGNTGGNC-GCCGNNNGG-CGCAAC 879

5-ct TGAT---GCTCATGTTTACCTGGCA-CCTGCGCAAGGCTGGTC-GCCGAANGGGCGCANC 879

7-ct TGAT---GCTCATGTTTACCTGGCA-CCTGCGCAAGGCTGGTC-GCCGAANGG-CGCAAC 876

11-ct TGAT---GCTCANGTTTACCTGGCA-CCTGCGCAAGGNTGGTC-GCCNAAGGN-NGCAAC 876

10-ct TGAT---GCTCATGTTTACCTGGNA-CCTGCGCAAGGNTNGTC-GCCGANGGG-CGCAAC 877

12-ct TGAT---GCTCATGTTTACCTGGCA-CCTGCGCAAGGNTGGTC-GCCNNNNNGGCGCAAC 879

9-ct TGAT---GCTCANGTTTACCNNNNNACCNGCNCAANGNTGGTC-GCCNANGGG-CNCAAC 879

3-ct TGAT---GCTCATGTTTACCTGGCA-CCNGCNCANNNNGNNNN-NCNGAAGGG-CGCNNC 881

1-ct NNNNATNNNTNANGTTNNCNNGNNNNNNNNNGNNNGNNNNNNNNNNNNNAAAGGNNNCAA 898

2-ct NNNNNNNNNNNNNNNTTNNCNGNNNNNNNNGNNNNGNNNGGNCGNCNGAANGGNNNNNAN 894

4-ct TGAT---GCTCNTGNTTANCNGGNNNNNNNNNNNNNGGNNGGNCNNCCNAAGGGCGCNNC 887

*

6-ct GC----AATATGGGCAGNATCGTCTGCATTTA-GCAGANNATTGCTGG-CATCGCTGA-C 931

8-ct GC----AATATGGGCAGGATCGTCTGCATTTA-GCAGATAATTGCTGG-CATCGCTGA-C 932

5-ct GC----AATATGGGCAGGANCGTCTGCATTTA-GCAGANNATTGCTGG-CATCGCTGA-C 932

7-ct GC----AATATGGGCAGGATCGTCTGCATTTA-GCAGATAATTGCTGG-CATCGCTGA-C 929

11-ct GC----ANTATGGGCAGGATCGTCTGCNTTTA-NCANATAATTGCTNG-CATCGCTGA-C 929

10-ct GC----NNTATGGGCAGGANCGTCTGCNTTTA-GCANATAANTGCTGG-CATCGCTGA-C 930

12-ct NC----NNTANGGGCAGGATCGTCTGNNTTTA-GCANATAANTGCTGG-CATCGCTGA-C 932

9-ct GC----NNTATGNGCAGGATCGTCNGNNTTTA-GCANANAATTGCTGG-CATCNCTNA-N 932

3-ct GC----ANTNNGGGNAGGANCGNCTGCATTTA-GCNNANAANTGCNNGGCATNNNNNNAN 936

1-ct CNGCNATNNNNGNNNNNNNNNNNNNNNNNTTTTNNNCNNANNATTNNNNNGTNTNNNTNN 958

2-ct NN--NANNANGNGCNNGAAANNNNNNCATTTNAGNNNAANANNNNCNGGCNTNNCNNNNN 952

4-ct NNN-ANNANNNGNNNGGNANCNTCTGCNTTTNNNNNNANNNNNCNNGNNNNNCNNNANNT 946

**

6-ct NNGCNNTTGCCCTNGTTTATCAATGCCCAGCANCACNACNTCGAAGCGACTTTTNNNNNA 991

8-ct GTGCCATTGCCCTTNNNN-TCAATGCCCAGCAGCACNACGTCGAANCGACTTTTANNAA- 990

5-ct GNGCCATTGCCCTTGTTTATCAATGCCCAGCAGCACAACGTCNAANCGACTTTTATNNN- 991

7-ct GTGCCNTTGCCCTNGTTTATCAATGCCCAGCAGCACAACGTCGAAGCGACTTTTATNNN- 988

11-ct NTGCCNTTGCCCNNGTTTATCAATGNCCAGCAGCANNANNTCGAANCNACTTTTNTCAA- 988

10-ct GTGNCNTTGCCCNTGTTNATCNANGNCCAGCAGCACNACNTCNAAGCGACTTTTNNCAA- 989

12-ct GTGCCATTGCCCTTGTTTNTCNATGNCCAGCAGCNCNANNTCGAAGCGANTTTTATCAA- 991

9-ct NTGNCNTTGCCCTNGTTNNTCAATGNCCAGCAGCNNNNNNTCNAANCGANTTTNANCAA- 991

3-ct NTNCCNTTGNNNTNGTTTNNNANNGCCCNNCAGCNNNACNNCNAANCNNNTTTNNNANT- 995

1-ct NNNTNCNNNTTNGCCNNNNNTNNNNNNNGNCNNNCANNNNNAACNNNNANNNNNNTTTTT 1018

2-ct NNNNNNNTGCCCNTGNNNANNNNNNNCNNNNNNNNNNNNNNNNNNNNNNNNNNNNNNNTN 1012

4-ct NCNNNNNNNNNTG-GTTNNNNANGNNCCNNNNANNNNNNNNNCNNAANNNNANTTTTNNN 1005

6-ct TGGNATCNACNANGNTTTTTGNCNATTGNNNANNNNNTTCATGNNCCCNCNNNNNNNCNC 1051

8-ct TGGNNTCGACNATGNTTTTTGNCGATTGCNNANACACTTCATGNTCCNNTGATTNNCCAC 1050

5-ct -NGNNTCNANNNGNTTTT--GNCGANTGCNNANNNNNTNNNTNNNNNN-TNANTTNCNAN 1047

7-ct -NGNNTCGANNNNNTTTTT-GNCNANTGCAGAGACNCTTNATGNTNCGCTGANTTNCNAN 1046

11-ct TGGNATCGACNATGTTTTTTGNCNATNGNNNANNNNNTTCNNGNTCNGCTGATTTNCCAN 1048

10-ct TGGCNTCGANNNNGTTTTTTGNCNATNGCNNANANNCT-----TCANGNTNNGCTNANTT 1044

12-ct TGNNNTCNANNNNGNTNTTTGNCNANNNNNANNNNCNTC-ANGNNNNNTNANNNNNNNNC 1050

9-ct NNGNNTCNANNATGTTNTTTGNCNANNGCNNANANNNTTNNNGNTCNGCTNANTTNCNAN 1051

3-ct NNNNTNNNNNNNNNNNNNTNGNCGNNNNGNNNNNNNNTTNNNGTTNCNNNNANTTNNNNN 1055

1-ct TNTNNTTGGNNNNNNNNNNNNNNNNTNTTNNNNCCNNCNNNNNNNACNANTCNNANNNNN 1078

2-ct NNNNNNNGNNNNNNNNNNNNNGNNNNNNNNNNANNACNATANANNANNNNANNNNANNNN 1072

4-ct NTNNNNNGNNNNNNNNNNNNNNNTNNNTNGGGCNNNNNNNNNNNNNNNNNNNNNNNTNNN 1065

*

6-ct CNAAANNNATTCNNNNCNNNNANTTTTNNNNNNNNCCCNNN----- 1092

8-ct CAAAANNNANTCCTACCNNNNANNTTTNNNCNNNNNNCCCNN---- 1092

5-ct CNAAANNNNNNNNNCNNNNANNTTNNNCNNNTNCCCNNNN------ 1087

7-ct NNAAANNNANTTNCNNNCNNCANNTTNNNNNNNNNNNCCCNNN--- 1089

11-ct CNAAAAANCGAANTNCNACCNNNANNNNNNN--------------- 1079

10-ct NCNANCAAAANNANNTNCNANCNNNANNNNN--------------- 1075

12-ct AAAAANNANNNNANCNNNANNNNNNNNNNNNNNNCNNNNNN----- 1091

9-ct CNAAANNNNNNCNNANNNNNANNNNNNNNNNNNNNNNCNNNNNNNA 1097

3-ct NNNNANNNNNNNNNNNNNCNNNN----------------------- 1078

1-ct CANNNNANNNNNNNNACACNNANANNNNANNNNNNNNNN------- 1117

2-ct NNNNNNNNNNNNNNNNNNCNNNNANNNN------------------ 1100

4-ct NNNNNNNTNNNNNNNNNNNNNCCNNNNNNNNNNNNNNN-------- 1103
