# Supplementary material for: Removal of PCR Error Products and Unincorporated Primers by Metal-Chelate Affinity Chromatography
Source: PLoS One. 2011 Jan 14;6(1):e14512. doi: 10.1371/journal.pone.0014512 (PMC3021510; doi:10.1371/journal.pone.0014512)
Supplement: Table S1 — Number of no-calls using CT primer. (0.03 MB DOC) [file pone.0014512.s006.doc]

Table S1: **Number of no-calls using CT primer**

| **Sample** | **Number of no-calls ‘N’s in the first 800 nt after X number of correctly called consecutive bases where X is equal to** | | | |
| --- | --- | --- | --- | --- |
|  | **0** | **5** | **10** | **15** |
| Unpurified1 | 99 | 50 | 50 | 50 |
| Unpurified2 | 111 | 64 | 64 | 64 |
| Unpurified3 | 52 | 10 | 10 | 10 |
| Unpurified4 | 73 | 27 | 27 | 27 |
| Unpurified Mean±SD | 84±26 | 38±24 | 38±24 | 38±24 |
| IMAC purified1 | 11 | 3 | 2 | 0 |
| IMAC purified2 | 14 | 4 | 1 | 1 |
| IMAC purified3 | 14 | 3 | 0 | 0 |
| IMAC purified4 | 22 | 7 | 4 | 4 |
| IMAC purified Mean±SD | 15±5 | 4±2 | 2±2 | 1±2 |
| QIAquick purified1 | 23 | 12 | 12 | 9 |
| QIAquick purified2 | 16 | 6 | 6 | 6 |
| QIAquick purified3 | 16 | 3 | 3 | 1 |
| QIAquick purified4 | 17 | 6 | 6 | 1 |
| QIAquick purified Mean±SD | 18±3 | 7±4 | 7±4 | 4±4 |
