# Supplementary material for: Removal of PCR Error Products and Unincorporated Primers by Metal-Chelate Affinity Chromatography
Source: PLoS One. 2011 Jan 14;6(1):e14512. doi: 10.1371/journal.pone.0014512 (PMC3021510; doi:10.1371/journal.pone.0014512)
Supplement: Table S2 — Number of no-calls using NT primer. (0.03 MB DOC) [file pone.0014512.s007.doc]

Table S2: **Number of no-calls using NT primer**

| **Sample** | **Number of no-calls ‘N’s in the first 800 nt after X number of correctly called consecutive bases where X is equal to** | | | |
| --- | --- | --- | --- | --- |
|  | **0** | **5** | **10** | **15** |
| Unpurified1 | 80 | 40 | 40 | 40 |
| Unpurified2 | 85 | 43 | 43 | 43 |
| Unpurified3 | 133 | 91 | 91 | 91 |
| Unpurified4 | 158 | 130 | 119 | 119 |
| Unpurified Mean±SD | 114±38 | 76±43 | 73±38 | 73±38 |
| IMAC purified1 | 19 | 5 | 3 | 3 |
| IMAC purified2 | 22 | 6 | 6 | 6 |
| IMAC purified3 | 18 | 3 | 1 | 1 |
| IMAC purified4 | 18 | 6 | 4 | 4 |
| IMAC purified Mean±SD | 19±2 | 5±1 | 4±2 | 4±2 |
| QIAquick purified1 | 40 | 24 | 22 | 22 |
| QIAquick purified2 | 34 | 18 | 18 | 18 |
| QIAquick purified3 | 26 | 9 | 7 | 7 |
| QIAquick purified4 | 24 | 9 | 7 | 7 |
| QIAquick purified Mean±SD | 31±7 | 15±7 | 14±8 | 14±8 |
